# PGC-1α mediates migrasome secretion accelerating macrophage–myofibroblast transition and contributing to sepsis-associated pulmonary fibrosis

**DOI:** 10.1038/s12276-025-01426-z

**Published:** 2025-04-01

**Authors:** Yawen Peng, Shuya Mei, Xiaohui Qi, Ri Tang, Wenyu Yang, Jinhua Feng, Yang Zhou, Xi Huang, Guojun Qian, Shunpeng Xing, Yuan Gao, Qiaoyi Xu, Zhengyu He

**Affiliations:** 1 Department of Critical Care Medicine, Ren Ji Hospital, Shanghai Jiao Tong University School of Medicine, Shanghai, China; 2 Key Laboratory of Anesthesiology (Shanghai Jiao Tong University), Ministry of Education, Shanghai, China; 3 Department of Cardiovascular Surgery, Ren Ji Hospital, Shanghai Jiao Tong University School of Medicine, Shanghai, China

**Keywords:** Cell migration, Cell polarity

## Abstract

Sepsis-associated pulmonary fibrosis (SAPF) is a critical pathological stage in the progression of sepsis-induced acute respiratory distress syndrome. While the aggregation and activation of lung fibroblasts are central to the initiation of pulmonary fibrosis, the macrophage–myofibroblast transition (MMT) has recently been identified as a novel source of fibroblasts in this context. However, the mechanisms driving MMT remain inadequately understood. Given the emerging role of migrasomes (novel extracellular vesicles mediating intercellular communication), we investigated their involvement in pulmonary fibrosis. Here we utilized a lipopolysaccharide-induced SAPF mouse model and an in vitro co-culture system of fibroblasts and macrophages to observe the MMT process during SAPF. We found that lipopolysaccharide exposure suppresses PGC-1α expression in lung fibroblasts, resulting in mitochondrial dysfunction and the accumulation of cytosolic mitochondrial DNA (mtDNA). This dysfunction promotes the secretion of mtDNA-containing migrasomes, which, in turn, initiate the MMT process and contribute to fibrosis progression. Notably, the activation of PGC-1α mitigates mitochondrial dysfunction, reduces mtDNA-migrasome release, inhibits MMT and alleviates SAPF. In conclusion, our study identifies the suppression of PGC-1α in lung fibroblasts and the subsequent release of mtDNA migrasomes as a novel mechanism driving MMT in SAPF. These findings suggest that targeting the crosstalk between fibroblasts and immune cells mediated by migrasomes could represent a promising therapeutic strategy for SAPF.

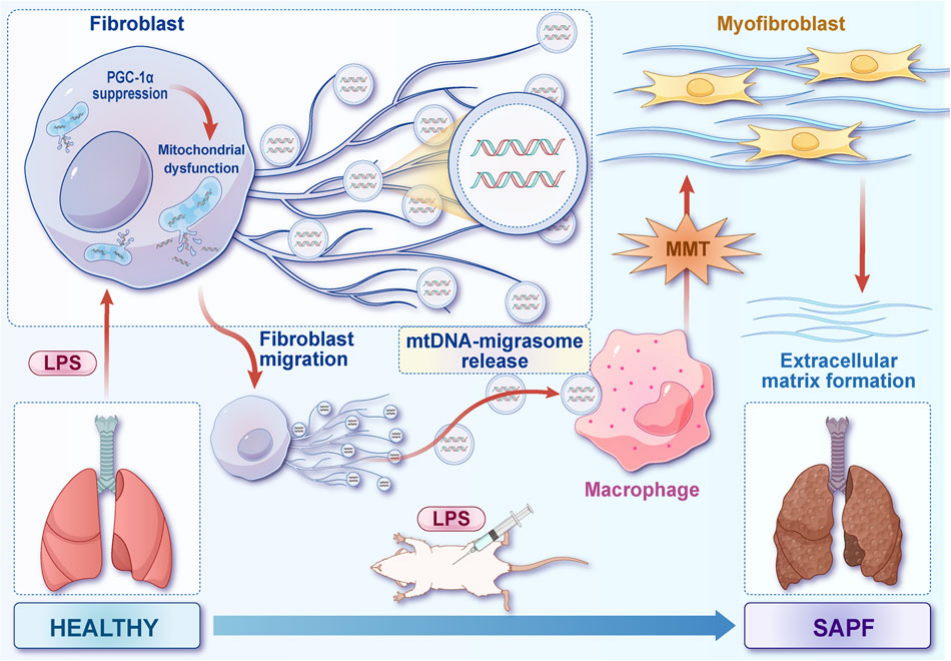

## Migrasomes drive fibrosis in sepsis-associated lung damage

Sepsis is a severe response to infection that can lead to lung damage, including pulmonary fibrosis. Researchers found that fibroblasts release small particles called migrasomes, which contain mitochondrial DNA (mtDNA) when exposed to lipopolysaccharide, a bacterial component. These migrasomes can be taken up by macrophages, causing them to transform into myofibroblasts (cells that contribute to fibrosis). The study used single-cell RNA sequencing (a method to analyze gene expression in individual cells) and other techniques to observe these changes. They discovered that activating a protein called PGC-1α in fibroblasts can reduce the release of mtDNA migrasomes, thereby decreasing fibrosis. This suggests that targeting PGC-1α could be a potential treatment for sepsis-related lung fibrosis. Future research may focus on understanding the detailed mechanisms of mtDNA’s role in fibrosis and developing therapies based on these findings.

This summary was initially drafted using artificial intelligence, then revised and fact-checked by the author.

## Introduction

Sepsis, a severe systemic inflammatory response to infection, is a leading cause of acute lung injury or acute respiratory distress syndrome (ARDS)^1^. Among the various complications of sepsis, sepsis-associated pulmonary fibrosis (SAPF) represents a critical pathological stage of sepsis-induced ARDS^2^. The acute inflammatory response to lipopolysaccharide (LPS), a component of the cell walls of Gram-negative bacteria, promotes fibroblast aggregation and the formation of fibroblastic foci^3^. These fibroblastic foci serve as the pathological basis for the initiation and progression of SAPF^4^. Understanding the mechanisms driving fibroblast activation and foci formation is therefore essential for developing novel therapeutic interventions for SAPF.

Myofibroblasts and fibroblast foci are hallmark features of various pulmonary fibrosis forms, including idiopathic pulmonary fibrosis and SAPF. Persistent activation of fibroblasts and the formation of fibroblast foci are key to pulmonary fibrosis progression. Emerging evidence suggests diverse cellular origins for these myofibroblasts, including bone-marrow-derived fibrocytes, endothelial-to-mesenchymal transition^5,6^, epithelial-to-mesenchymal transition^7^ and hyalocyte transdifferentiation^8^. Macrophages are a pivotal cell type in fibrotic disease development^9^. Recent studies have identified macrophage–myofibroblast transition (MMT) as a crucial mechanism contributing to fibrosis^10–13^. These observations suggest that MMT may be another pathway for myofibroblast accumulation in pulmonary fibrosis. The emerging single-cell RNA sequencing (scRNA-seq) technique, which allows the analysis of cell-to-cell transcriptome profiles on a genomic scale, has led to profound discoveries in biology^14,15^. In this study, utilizing scRNA-seq technology, we have identified the MMT phenomenon, revealing a novel mechanism for the generation of pathogenic fibroblasts in SAPF. However, the molecular mechanisms underlying MMT in SAPF remain poorly understood.

Migrasomes, a newly identified subtype of released cell fragments, play a important role in cell migration and intercellular communication^16^. These large vesicles (0.5–3 μm in diameter), which are formed along retraction fibers during cell migration, facilitate the transfer of cellular contents including mitochondria, proteins and RNA between migrating cells^17^. This transfer may coordinate collective cell migration, promoting cell polarity and directionality. The activation and differentiation of fibroblasts into myofibroblasts are critical processes in the development of pulmonary fibrosis. Migrasomes could enhance intercellular communication within the fibrotic lung microenvironment, potentially transferring profibrotic factors such as transforming growth factor-β (TGF-β), which strongly induces fibroblast activation and extracellular matrix production^18^. This suggests that migrasomes released by activated fibroblasts may modulate the behavior of epithelial cells, endothelial cells and immune cells, contributing to fibrosis progression. However, the role of migrasomes in the SAPF process, particularly their secretion mechanisms and downstream effects, remains unclear.

Mitochondria are essential for maintaining cellular health and survival, with their homeostasis being crucial throughout their lifecycle^19^. Peroxisome proliferator-activated receptor gamma co-activator 1-α (PGC-1α), a transcriptional co-activator that regulates mitochondrial biogenesis, has been implicated in lung injury and repair^20^. Previous studies have demonstrated that PGC-1α-deficient mice are more susceptible to bleomycin-induced lung fibrosis, suggesting its potential involvement in lung pathology^21^. Furthermore, mitochondrial DNA (mtDNA), the sole form of extranuclear double-stranded DNA (dsDNA) in eukaryotic cells, notably initiates inflammation when released from damaged mitochondria^22^. Elevated levels of mtDNA in plasma are linked to ARDS development in patients with trauma and sepsis^23^. However, the role of PGC-1α in controlling fibroblast migrasome release and its impact on macrophage behavior has not been elucidated.

In this study, we hypothesized that migrasomes secreted by fibroblasts during sepsis facilitate MMT and contribute to SAPF by mediating crosstalk between mesenchymal cells and immune cells. Using an in vitro co-culture system and LPS-induced SAPF mouse model, we investigated the mechanisms underlying MMT. Specifically, we examined the expression and activation status of PGC-1α and explored the role of mitochondrial dysfunction and mtDNA in promoting migrasome-mediated intercellular communication. Our findings illuminate the complex mechanisms underlying SAPF pathogenesis and highlight the interplay between mesenchymal cells and immune cells, mediated by novel organelle termed migrasomes, as a potential therapeutic target for this debilitating condition.

## Material and methods

Key resource details are described in Table 1.

**Table 1:** Key resource details.

Source	Catalog	Reagent or resource
**Antibody**		
Abcam	ab150115	Goat anti-mouse IgG H&L (Alexa Fluor 647)
Abcam	ab150160	Goat anti-rat IgG H&L (Alexa Fluor 594)
Abcam	ab150105	Donkey anti-mouse IgG H&L (Alexa Fluor 488)
Abcam	ab150114	Goat anti-mouse IgG H&L (Alexa Fluor 555)
Abcam	ab27156	Anti-ds DNA antibody (35I9 DNA) – BSA and azide free
Abcam	ab270993	Recombinant anti-collagen I antibody (EPR24331-53)
Abcam	ab252432	Recombinant anti-mtTFA antibody (EPR23548-120)
Abcam	ab201693	Recombinant anti-PIGK antibody (EPR17843)
Abcam	ab191838	Anti-PGC1-α antibody
Abcam	ab5694	Anti-α smooth muscle actin antibody
Abcam	ab6640	Anti-F4/80 antibody (CI:A3-1)
Abcam	ab7817	Anti-α smooth muscle actin antibody (1A4)
Abcam	ab119857	Anti-CD86 antibody (GL-1)
Abcam	ab64693	Anti-mannose receptor antibody
ABClone	AC026	β-Actin rabbit mAb
ABClone	A10253	TSPAN4/NAG-2 rabbit pAb
ABClone	A19069	Integrin alpha 5 (ITGA5/CD49e) rabbit mAb
BD Pharmingen	564406	Fixable viability stain 510
BD Pharmingen	570293	CD11b RB705 M1/70
Biolegend	103115	APC/cyanine7 anti-mouse CD45 antibody (30-F11)
Biolegend	123115	APC anti-mouse F4/80 antibody (BM8)
Biolegend	101205	FITC anti-mouse/human CD11b antibody
Biolegend	141705	PE anti-mouse CD206 (MMR) antibody (C068C2)
Biolegend	105013	PE/cyanine7 anti-mouse CD86 antibody(GL-1)
Biolegend	137013	PE anti-mouse CD68 antibody
Novus	NBP2-34522AF405	α-SMA antibody (1A4/asm-1) (Alexa Fluor 405)
SouthernBiotech	0100-20	DAPI Fluoromount-G
SouthernBiotech	0100-01	Fluoromount-G
Proteintech	66240-1-Ig	β-Tubulin monoclonal antibody
Proteintech	26203-1-AP	NDST1 polyclonal antibody
Beyotime	A0208	Anti-rabbit IgG, HRP-linked antibody
Beyotime	A0216	Anti-mouse IgG, HRP-linked antibody
**Chemicals, peptides and recombinant proteins**		
MCE	HY-15687A	SAR407899
MCE	HY-17538	ZLN005
Sigma	F2006	Fibronectin human plasma
Sigma	L2630	LPS from *Escherichia coli* O111:B4
Sigma	L4516	LPS from *Escherichia coli* O127:B8
**Critical commercial assays**		
Abcam	ab65321	mtDNA isolation kit
invitrogen	M7512	MitoTracker Red CMXRos
invitrogen	L3000015	Lipofectamine 3000
invitrogen	W7024	WGA sampler kit
Invitrogen	W32466	WGA
Miltenyi	130-095-927	Lung dissociation kit, mouse
Sigma	LYSISO1	Lysosome isolation kit
Qiagen	69504	DNeasy Blood & Tissue Kit (50)
BD Pharmingen	554714	Fixation/permeablization kit
Pierce	23227	BCA protein assay kits
BD Pharmingen	555899	Lysing buffer
Yeasen	40203ES76	CCK-8
Vazyme	Q411-02/03	ChamQ SYBR Color qPCR Master Mix
**Experimental models: cell lines**		
Raw264.7		
L929		
TSPAN4-mCherry-L929		
TSPAN4-mScarlet-PGC-1α-ZsGreen L929		
**Primers**		
Sangon Biotech	Mouse D-loop	Forward: AATCTACCATCCTCCGTGAAACC
		Reverse: TCAGTTTAGCTACCCCCAAGTTTAA
	Mouse Cox1	Forward: GCCCCAGATATAGCATTCCC
		Reverse: GTTCATCCTGTTCCTGCTCC
	Mouse ND1	Forward:CTAGCAGAAACAAACCGGGC
		Reverse:CGGCTGCGTATTCTACGTT
	Mouse 18S	Forward: TAGAGGGACAAGTGGCGTTC
		Reverse: CGCTGAGCCAGTCAGTGT
	Mouse B2m	Forward: ATGGGAAGCCGAACATACTG
		Reverse: CAGTCTCAGTGGG GGTGAAT
**Other**		
Labselect	14122	Transwell-Clear inserts, membrane pore size 3.0 μm, polyester (PET) membrane
Labselect	14112	Transwell-Clear inserts, membrane pore size 0.4 μm, polyester (PET) membrane
Gibco	C11875500BT	RPMI 1640
Gibco	11995065	DMEM
Gibco	15140122	Penicillin–streptomycin
Gibco	12604013	TrypLE Express
**Software and algorithms**		
GraphPad Software	Prism 10	N/A
ImageJ	ImageJ	N/A
FlowJo	FlowJo 10	N/A

N/A, not applicable.

### Animal studies

All animal experimentation protocols were approved by the Ren Ji Hospital Ethics Committee of Shanghai Jiao Tong University School of Medicine (approval no. RJ2022-0610). C57BL/6 male mice were purchased from Shanghai SLAC Laboratory Animal, China, and kept in conditions free of specific pathogens at 22 ± 2 °C with 40–60% humidity, diurnal lighting and ad libitum access to food and water. Six- to 8-week-old male wild-type mice were allocated randomly and subjected to LPS-induced SPF as described^24^. In brief, 5 mg/kg LPS or vehicle was intraperitoneally injected for three consecutive days, and the mice were euthanized 7 days later. In the PGC-1α-overexpressed mouse model, adeno-associated virus (AAV) overexpressing PGC-1α was acquired from Shanghai Genechem. Mice received intratracheal administration of capsid AAV9 (CAG-MCS-3flag-T2A-EGFP-SV40 PolyA) or PGC-1α AAV (5 × 10^11^ vg in 50 µl of saline per mouse) 3 weeks before LPS injection. Mice were euthanized 1 week after LPS administration, after which serum was collected to carry out the next experiments. The lungs were inflated with cold phosphate-buffered saline (PBS), excised for flow cytometry and western blot, and fixed in 4% paraformaldehyde for histological evaluation. Schematic illustrations of animal experimental designs are shown in Supplementary Figs. 1a and 7a.

### Cell lines

L929 and Raw264.7 cell line were acquired from Shanghai Fuheng Biology. TSPAN4-mCherry-L929 stable cell lines were generously provided by Prof. Li Yu (Tsinghua University). Cells were cultured in Dulbecco’s modified Eagle medium (DMEM) supplemented with 10% fetal bovine serum (Gibco) and 100 IU/ml penicillin–streptomycin in 5% CO_2_ at 37 °C.

To generate the stable PGC-1α-overexpressing L929 cell line, PGMLV-CMV-Ppargc1a-3× Flag-EF1-ZsGreen1-T2A-Puro plasmid was acquired from Shanghai Genomeditech, L929 cells were transfected with lentivirus and then the cells were selected in medium containing 2 µg/ml puromycin. The transfection efficiency was confirmed by fluorescence microscopy and western blotting (Supplementary Fig. 6a, b). To generate the TSPAN4-mScarlet L929 stable cell line, lenti-CMV-Mouse_Tspan4-mScarlet-PGK-Blasticidin plasmid was acquired from Shanghai Genomeditech, and L929 cells were transfected with lentivirus and then selected with 2 µg/ml blasticidin in medium. The transfection efficiency was confirmed by fluorescence microscopy as shown in Supplementary Fig. 6c.

For LPS stimulation, 1 μg/ml LPS was exposed to L929 for 48 h, while the same volume of PBS was used in the control group. The ZLN005-challenged cells were pretreated with ZLN005 for 24 h before LPS exposure.

### CCK-8 assay

Cell viability was evaluated by Cell Counting Kit 8 (CCK-8) according to the manufacturer’s protocols. Cells were seeded and cultured at a density of 2 × 10^4^ per well in 100 μl of medium into 96‐well microplates (Corning). Then, the cells were treated with various concentrations of ZLN005 (0, 1, 5, 10, 25 and 50 μM) for 24 h. All experiments were performed in triplicate. After incubation with the CCK-8 solution for 2 h, the absorbance was analyzed at 450 nm using a microplate reader, with wells without cells as blanks. The cell viability was expressed by the absorbance.

### Migrasome purification

Migrasome purification was performed using iodixanol–sucrose density-gradient centrifugation^16^. In brief, cells were seeded on 150-mm cell culture dishes with 1 μg/ml fibronectin pretreated for 2 h at 37 °C. Usually, at least 30 dishes are required to purify one batch of migrasomes. The first step is designed to remove cell bodies and large cell debris by centrifugation at 1000–4000*g*. After this, the pellet was discarded and the supernatant was centrifuged at high speed (20,000*g*) to pellet the migrasomes. Subsequently, the migrasome-containing pellet was resuspended and subjected to density-gradient centrifugation (lysosome isolation kit; Sigma-Aldrich, cat. no. LYSISO1) at 150,000*g*. The final ultracentrifuged product was divided into several fractions (migrasomes are usually enriched in fractions 3–5), and then the migrasome fraction was washed in an equivalent volume of PBS and spun down at 20,000*g* to obtain the migrasome pellet. The migrasome pellet was used for western blot and electron microscopy observation.

### Detection and observation of migrasomes in cell

In this study, the L929 cell line was chosen to observe the migrasome, and the procedure was based on a modification of previous protocols^17^. Here, 35-mm confocal dishes were precoated with fibronectin (100 μg/ml) at 37 °C for 12 h. Cells were cultured in fibronectin-precoated confocal dishes for 10–12 h, then treated with serum-free DMEM medium with or without LPS (1 μg/ml) for 48 h. For imaging, stained migrasomes with wheat germ agglutinin (WGA) (5 μg/ml) were observed using a confocal microscopy system (Olympus FV3000).

### Optimization of migrasome concentration for macrophage stimulation

First, migrasomes from fibroblasts of different groups were extracted, purified and quantified using a BCA protein assay. The purified migrasomes were then added to the macrophage culture medium and incubated for 24 h. To determine the optimal migrasome concentration for macrophage stimulation, four concentration gradients (0.1, 0.5, 1 and 2 µg) were tested (data are shown in Supplementary Fig. 4b). Ultimately, a migrasome protein concentration of 0.1 µg was selected for further experiments.

### Transmission electron microscopy

For the morphological analysis of purified migrasomes and mouse lung tissues, samples were fixed with 2.5% glutaraldehyde in phosphate buffer for 2 h at room temperature, washed three times with phosphate buffer and then post fixed with 1% osmium tetroxide for 1 h at room temperature. All samples were then dehydrated with a graded series of ethanol concentrations (30%, 50%, 70%, 90%, 95% and 100%) for 8 min each. Samples were infiltrated with and embedded in Epon812 resin. After polymerizing sequentially for 6 h at 37 °C, 12 h at 45 °C and 24 h at 60 °C, respectively, 70-nm-thick ultrathin sections were cut using a diamond knife. The sections were double stained with uranyl acetate and lead citrate. After air drying, the samples were examined with a transmission electron microscope H-7800 (Hitachi) at an acceleration voltage of 80 kV.

### Immunofluorescence assay

Lung sections were rinsed and blocked after antigen retrieval. Primary antibodies, including F4/80, CD86, CD206 and α-SMA, were incubated overnight at a dilution of 1:100 at 4 °C. After triple rinses, the sections were incubated with Alexa Flour 488, Alexa Flour 555, Alexa Flour 594 and Alexa Flour 647 for 60 min inside a humidity chamber. The slides were rinsed and then mounted using medium that contained 4′,6-diamino-2-phenylindole (DAPI). The covered slides were examined by a fluorescence microscope (Leica).

The mitochondrial morphology in L929 cells was analyzed with MitoTracker Red according to the manufacturer’s instructions. The samples were then fixed in 4% paraformaldehyde, permeabilized in 0.3% Triton X-100 and blocked in 1× PBS supplemented with 5% bovine serum albumin (BSA). Primary antibodies were incubated in blocking buffer at 4 °C overnight. Secondary Alexa antibodies were added for 1 h. Nuclei were counterstained with DAPI. Samples were imaged through an Olympus FV3000 confocal microscope.

### Immunohistochemistry

Lungs were collected and immediately fixed in 4% paraformaldehyde, sectioned into 5 μm thickness and stained with hematoxylin and eosin (H&E) to evaluate gross morphology and lung damage and inflammation, and Masson’s trichrome staining was performed to evaluate pulmonary collagen deposition.

### Real-time PCR

mtDNA was quantified by real-time PCR, which was performed using real-time PCR reagents on a LightCycler 480 real-time PCR System (Roche) and calculated by the 2^−ΔΔCT^ method. Primer sequences were obtained from the NIH qPrimerDepot (http://mouseprimerdepot.nci.nih.gov) and provided by Sangon Biotech.

### Measurement of total mtDNA

Total DNA of mouse lung tissues, purified migrasomes, fibroblasts primed with LPS for 48 h and macrophages co-cultured with fibroblasts were isolated using the DNeasy Blood & Tissue Kit according to the manufacturer’s instructions. mtDNA was quantified by real-time PCR using primers specific for the mitochondrial D-loop region, cytochrome oxidase subunit 1 (Cox1) or NADH dehydrogenase 1 (ND1). Nuclear DNA encoding 18S ribosomal RNA and β2 microglobulin (B2m) was used for normalization.

### Cellular fractionation and measurement of cytosolic mtDNA

Mitochondrial fractionation was performed using a mtDNA isolation kit (Abcam) according to the manufacturer’s instructions. In brief, cells were washed with PBS and collected, and 50% were saved for DNA extraction of whole cells. The remaining cells were resuspended with cytosol extraction buffer and incubated on ice for 10 min. Cells were homogenized in an ice-cold Dounce tissue grinder 80–100 times on ice. The homogenized cells were centrifuged at 1,200*g* for 10 min at 4 °C. The supernatant was further centrifuged at 10,000*g* for 30 min at 4 °C to pellet the mitochondria from the supernatant cytosolic fraction. Real-time PCR was performed after DNA purification with DNeasy Blood & Tissue Kit according to the manufacturer’s instructions, from both whole-cell extracts and cytosolic fractions using mtDNA primers (D-loop, Cox1 and ND1), or after DNA purification from whole-cell extracts using nuclear DNA primers (18S and B2m). The Ct values obtained for mtDNA abundance in whole-cell extracts served as normalization controls for the mtDNA values obtained from the cytosolic fractions.

### Preparation of mtDNA and stimulation of naive macrophage by cell-free mtDNA

Mitochondrial mtDNA from donor fibroblasts was isolated using a mtDNA isolation kit (Abcam) according to the manufacturer’s instructions. The eluted mtDNA was resuspended in 20 μl Tris-EDTA (TE) buffer and quantified using spectrophotometric analysis at 260/280 nm with a NanoDrop spectrophotometer. The amounts of mtDNA purified from different treatment groups varied. The control donor fibroblast secreted approximately 90 ng of mtDNA. The mtDNA amount secreted from LPS-primed donor fibroblast was low, approximately 60 ng. Fibroblasts overexpressing PGC-1α released about 100 ng of mtDNA, while LPS-primed, PGC-1α-overexpressing donor fibroblasts secreted approximately 110 ng. Recipient macrophages were transfected with 50 ng of the prepared mtDNA using Lipofectamine 3000 according to the manufacturer’s instructions.

### Western blot

Lung tissue extracts or cell lysates were prepared in RIPA buffer containing a protease inhibitor cocktail and a phosphatase inhibitor cocktail. Protein concentrations were determined using a BCA protein assay kit. Equal amounts of protein were separated by SDS–PAGE and transferred onto polyvinylidene difluoride membranes, blocked in 5% BSA in 1× TBST for 30 min and incubated with primary antibodies at 4 °C overnight. Secondary antibodies were added for 1 h, and detection was performed using the ChemiDoc imaging system.

### Flow cytometry analysis

The whole pulmonary tissue was enzymatically digested using a pulmonary dissociation kit (Miltenyi Biotec). The gentle MACS dissociations were used for mechanical dissociation samples; after that, the cell suspension was applied to a 70-µm filter and the cell suspension was centrifuged. Next, the supernatant was aspirated completely and then the cells were resuspended with appropriate buffer for further application. Then, the single-cell suspension was incubated with fluorochrome-conjugated antibodies for FVS510, CD45, F4/80, CD11b and CD86. After the cell surface staining procedure was completed, cells were fixed and permeabilized using the BD Cytofix/Cytoperm Fixation/Permeabilization Kit and stained with fluorochrome-conjugated antibodies of CD68, CD206 and α-SMA for 30 min. After washing with PBS, cells were analyzed by FACS Verse flow cytometry (BD). Data were analyzed with the FlowJo (version 10) software.

### scRNA-seq

scRNA-seq was conducted on lung cells obtained from mice treated with saline or LPS, which were prepared and preprocessed according to the manufacturer’s protocol of the 10x Genomics Chromium (Biomarker Technologies). Cellular suspensions were loaded on the Chromium Controller (10x Genomics) to generate gel bead-in-emulsions. Barcoded sequencing libraries were performed using Chromium Single Cell 3′ Reagent Kits v3.1 (10x Genomics), according to the manufacturer’s instructions. After the library preparation, the sequencing was performed with paired-end sequencing of 150 nt each end on one lane of NovaSeq 6000 per sample. Raw reads were processed using the 10x Genomics Cell Ranger pipeline (https://support.10xgenomics.com/single-cell-gene-expression/software/downloads/latest) with the mm10 as the reference. Cell Ranger can cluster the single cells, identify the marker genes of each cluster and export a matrix with unique molecular identifier values of each gene in a single cell. The R package SoupX and the scDblFinder procedure were used for quality control. The Scanpy package was used for further analysis and default parameters. The Mouse Cell Atlas (MCA) dataset and the corresponding cluster annotation were downloaded from MCA Gallery (https://bis.zju.edu.cn/MCA/index.html). By using cell clustering and annotation, fibroblasts and macrophages were identified, and velocyto and scVelo packages were used for downstream RNA velocity analysis^25,26^.

### Quantification and statistical analysis

Statistical parameters and significance are reported in the figures and the figure legends. Statistical analyses were conducted using the unpaired two-tailed *t*-test, one-way analysis of variance (ANOVA) and two-way ANOVA with Tukey’s multiple comparisons post-hoc test in GraphPad Prism 10 software (GraphPad Software). Log-rank (Mantel–Cox) test was used for survival analysis. The error bars represent mean ± s.d. Significance is indicated by asterisks: *P < 0.05, **P < 0.01, ***P < 0.001, ****P < 0.0001, and NS indicates no significance.

## Result

### MMT involved in LPS-induced pulmonary fibrosis

SAPF mouse models were established as shown in Supplementary Fig. 1a. Pulmonary injury and collagen deposition were assessed using H&E and Masson staining, showing significant interstitial leukocyte infiltration, alveolar edema and increased collagen deposition in the pulmonary interstitium in LPS-treated mice compared with controls (Supplementary Fig. 1b). Protein expression of extracellular matrix markers, collagen I and α-smooth muscle actin (α-SMA) were also upregulated after LPS administration (Supplementary Fig. 1c).

**Fig. 1:**
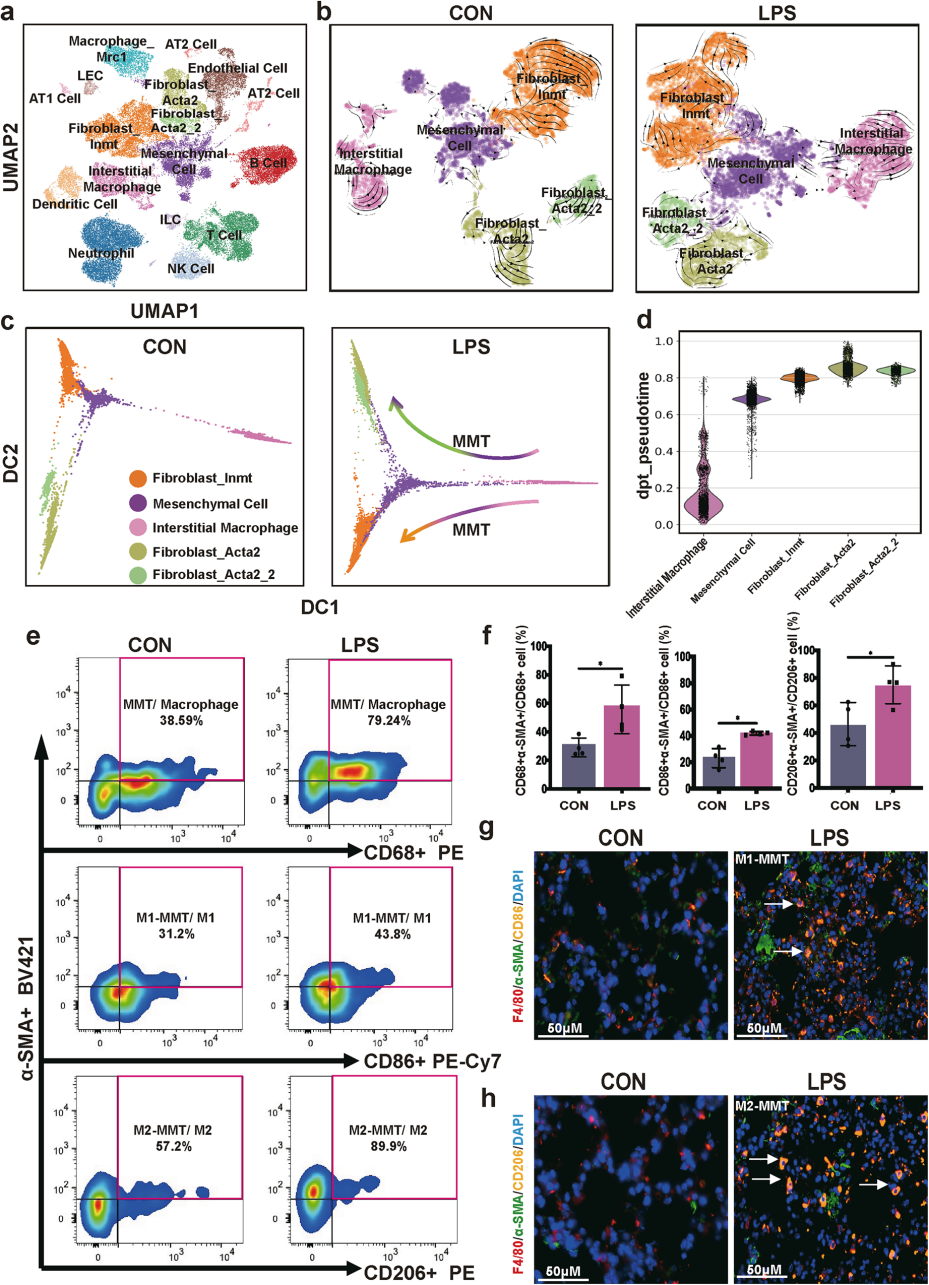
MMT involved in LPS-induced pulmonary fibrosis. **a** UMAP and clustering based on RNA expression data of mouse lung tissue. ILC, innate lymphoid cell; LEC, lymphatic endothelial cell. **b** RNA velocity analysis of fibroblast, mesenchymal cell and macrophage clusters of the control group and the LPS group respectively. **c** Pseudotime expression trends of fibroblast, mesenchymal cell and macrophage clusters over the pseudotime trajectory. **d** Violin plots of pseudotime of macrophage, mesenchymal cell and fibroblast clusters sorted by pseudotime. **e** MMTs (α-SMA^+^ CD68^+^), M1-MMTs (α-SMA^+^ CD86^+^) and M2-MMTs (α-SMA^+^ CD206^+^) in murine lung quantified by flow cytometry analysis. **f** Bar plots representing the percentage of α-SMA^+^ CD68^+^ cells to CD68^+^ cells, percentage of α-SMA^+^ CD86^+^ cells to CD86^+^ cells and percentage of α-SMA^+^ CD206^+^ cells to CD206^+^ cells in murine lung quantified by flow cytometry analysis (*P < 0.05, unpaired *t*-test, *n* = 4). **g** Multiplex immunofluorescence enlarged merged images of F4/80^+^ CD86^+^ α-SMA^+^ cells in mouse lung tissues. The arrows indicate the positive co-localized cell. Scale bar, 50 μm. **h** Multiplex immunofluorescence enlarged merged images of F4/80^+^ CD206^+^ α-SMA^+^ cells in mouse lung tissues. The arrows indicate the positive co-localized cell. Scale bar, 50 μm.

Given the pivotal role of macrophages in mediating inflammation and fibrosis in fibrotic diseases and the unclear role of MMT in SAPF^9^, we investigated the characteristics of MMT at the transcriptomic level using scRNA-seq on lung tissues from LPS-induced SAPF mice. Single-cell analysis allows the direct correlation of traditional markers of macrophage programing with cell function^27^. Uniform Manifold Approximation and Projection (UMAP) clustering is shown in Fig. 1a, with three representative marker genes for each cluster in Supplementary Fig. 2a. From UMAP analysis, we noted a close association between fibroblasts and macrophages. Consequently, we performed trajectory inference based on RNA velocity of whole lung cells from all mice, confirming the presence of MMT in the RNA-velocity-projected UMAP (Supplementary Fig. 2b). RNA velocity analysis of macrophage, mesenchymal cell and fibroblast clusters from control and LPS-challenged mice is shown in Fig. 1b, and the top ten driver genes are presented in Supplementary Fig. 2c. These results suggest that MMT occurs under LPS-induced pulmonary fibrosis but is absent in naive mice.

Pseudotime analysis revealed the developmental trajectory of macrophages starting from the CD68^+^ α-SMA^−^ cluster (pink) transitioning to mesenchymal cells (α-SMA^+^ CD68^+^, violet) and culminating at fibroblast clusters (CD68^−^ α-SMA^+^, green and orange) (Fig. 1c, d). This process was observed in LPS-induced SAPF mouse lungs but not in normal lung tissue. Consistent with our hypothesis, flow cytometry analysis detected an increase in α-SMA^+^ CD68^+^ cells in LPS-treated mice compared with controls (Fig. 1e, f). The macrophage gating strategy is shown in Supplementary Fig. 3a, and α-SMA^+^ and CD206^+^ populations were identified using fluorescence-minus-one controls (Supplementary Fig. 3b).

Due to macrophage phenotype, which is broadly stratified into classically activated or M1, which expresses the cell surface marker CD86, and alternatively activated or M2, with the cell surface marker CD206 (ref. ^9^), to validate which type of macrophage participated in the MMT process, we also detected these two different types of macrophage MMT situation. Interestingly, both M1-MMT (α-SMA^+^F4/80^+^ CD86^+^) and M2-MMT (α-SMA^+^F4/80^+^CD206^+^) populations (Fig. 1e, f) were significantly increased in the SAPF model. Furthermore, multiplex immunofluorescence imaging confirmed the presence of these MMT cells, both M1 and M2 subtypes, also expressing α-SMA and F4/80, exhibiting spindle-like myofibroblast morphology in SAPF mice, but absent in normal lung tissue (Fig. 1g, h and Supplementary Fig. 3c, d). These findings highlight the contribution of MMTs to the development of fibroblast foci in SAPF.

### LPS stimulation enhances fibroblast migration and migrasome release

**Fig. 2:**
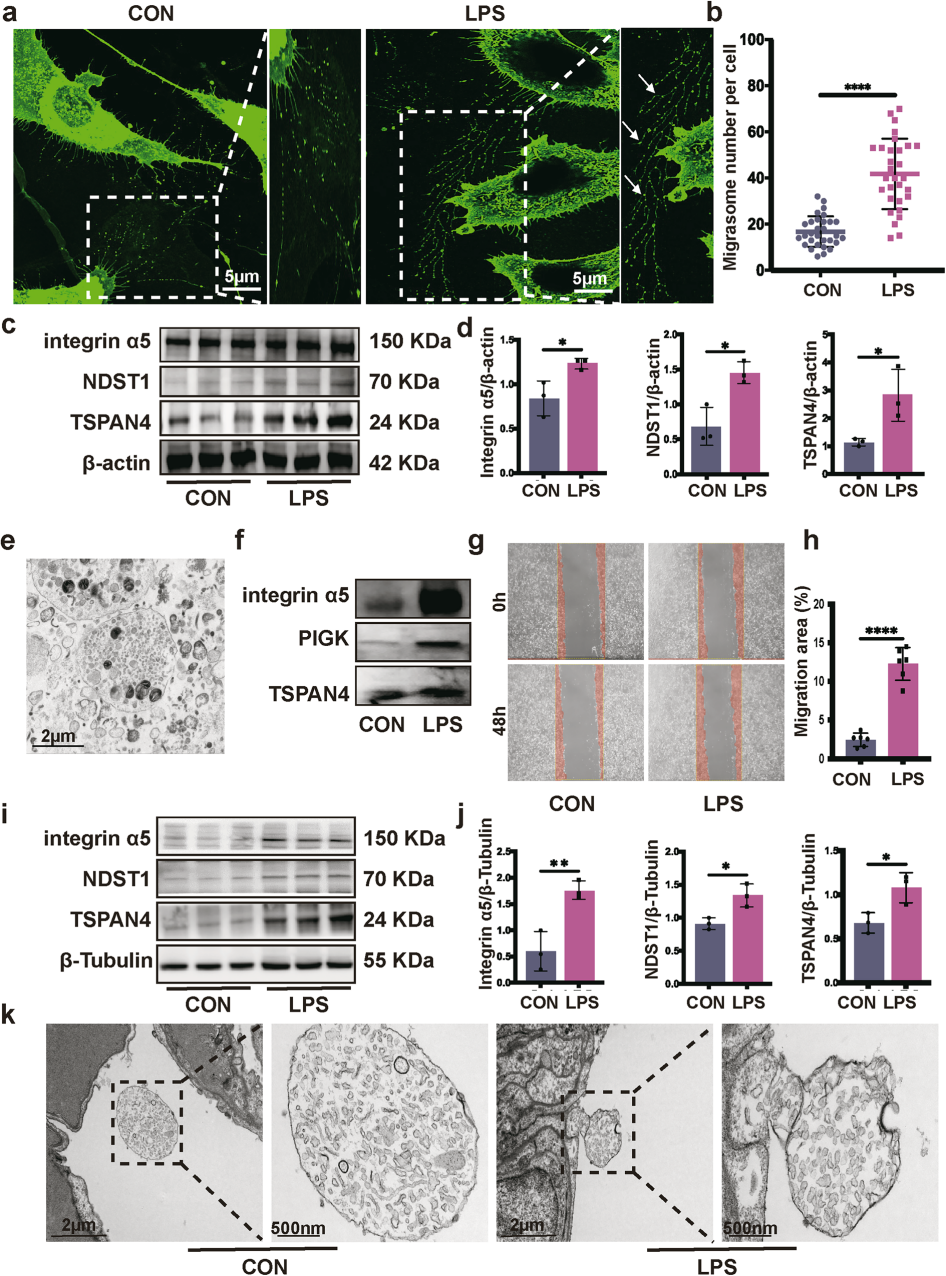
LPS stimulation increases fibroblast migrasome release in vitro and in vivo*.* **a** A typical confocal image of fibroblasts with WGA staining. Enlarged migrasomes are shown in the right panels. The white arrows show migrasomes. Scale bar, 5 µm. **b** Statistics of migrasome number per cell in migrasome-containing fibroblasts (****P < 0.0001, unpaired *t*-test, *n* = 30). **c**, **d** The protein expression of migrasome-specific markers integrin α5, NDST1 and TSPAN4 in fibroblasts was determined using western blot (**c**). The bar plots (**d**) show the relative expression (*P < 0.05, unpaired *t*-test, *n* = 3). **e** Purified migrasomes derived from fibroblasts were determined using TEM analysis. **f** Purified migrasomes derived from fibroblasts were determined using western blot. **g**, **h** Microscopic inspection (**g**) of L929 fibroblasts with or without LPS stimulation after scratching (0 h) and after 48 h of wound healing. The bar plots (**h**) show the relative fibroblast migration area percentage (*P < 0.05, ****P < 0.0001, unpaired *t*-test, *n* = 6). **i**, **j** The protein expression of migrasome-specific markers integrin α5, NDST1 and TSPAN4 in mouse lung was determined using western blot (**i**). The bar plots (**j**) show the relative expression (*P < 0.05, **P < 0.01, unpaired *t*-test, *n* = 3). **k** TEM images of the migrasome observed in normal and fibrotic mouse lung tissues. Scale bar, 2 µm. Enlarged migrasomes are shown in the right panels. Scale bar, 500 nm.

The differentiation of fibroblasts into myofibroblasts, which possess secretory, contractile and extracellular-matrix-producing properties, is a critical cellular event in the development of pulmonary fibrosis^26^. Previous studies have demonstrated a strong correlation between fibroblast migration and the progression of pulmonary fibrosis^4,27^. To investigate the mechanisms underlying fibroblast migration in fibrosis development, we treated the L929 fibroblast cell line with LPS. After 48 h of LPS treatment, there was a notable increase in expressions of collagen I and α-SMA in fibroblasts compared with the control group (Supplementary Fig. 4a). During fibroblast migration, the formation of migrasomes, characterized by the presence of terminal or junction of recontraction filament along with multiple vesicles^16^, was notably increased under LPS challenge (Fig. 2a, b). Furthermore, we observed a significant rise in the protein expression of migrasome-specific markers^28^—integrin α5, NDST1 and TSPAN4 in fibroblasts (Fig. 2c, d). Transmission electron microscopy (TEM) analysis revealed the presence of a few mitochondria within the purified migrasomes (Fig. 2e), and purified migrasomes were further confirmed via western blot using migrasome-specific markers (Fig. 2f). Wound healing assays also indicated a significant enhancement in fibroblasts migration capability after LPS stimulation (Fig. 2g, h).

To determine whether migrasomes are also present in the SAPF mouse model, western blot analysis of lung tissues from LPS-stimulated mice showed a significant increase in the expression of migrasome-specific markers integrin α5, NDST1 and TSPAN4 (Fig. 2i, j). Moreover, TEM analysis confirmed the presence of migrasome-like structure in mouse lung (Fig. 2k). These findings suggest that LPS-stimulated fibroblast migration may play a role in fibroblast–macrophage interactions that propagate the profibrotic niche associated with MMT.

### Fibroblast-released mtDNA migrasomes contribute to MMT in LPS-induced pulmonary fibrosis

**Fig. 3:**
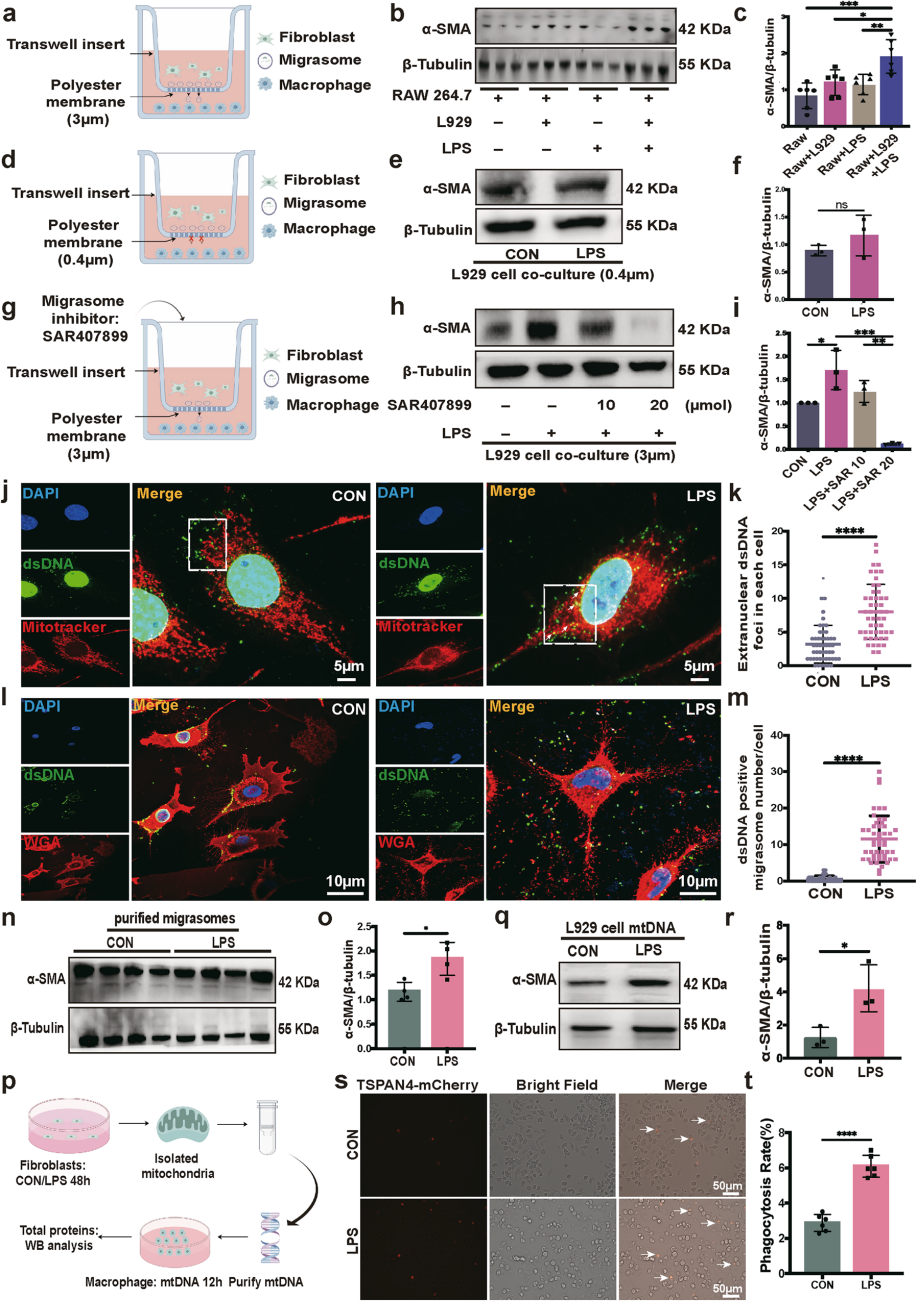
Fibroblast-released mtDNA migrasomes contribute to MMT in LPS-induced pulmonary fibrosis. **a** The experimental scheme of fibroblast and macrophage co-culture using transwell inserts with a pore size of 3 µm. **b**, **c** Immunoblotting images of the α-SMA protein in macrophages co-cultured with fibroblasts with or without LPS stimulation (**b**) The bar blots (**c**) show the relative α-SMA protein expression (*P < 0.05, **P < 0.01, ***P < 0.001, one-way ANOVA, *n* = 6). **d** The experimental scheme of fibroblast and macrophage co-culture using transwell inserts with a pore size of 0.4 μm to inhibit migrasomes’ access to the lower chamber. **e**, **f** Immunoblotting images (**e**) and analysis of the relative α-SMA protein expression in macrophages (**f**) which co-cultured with fibroblasts with or without LPS stimulation under obstruction of migrasome access to the lower chamber (transwell inserts with a pore size of 0.4 µm; NS, not significant, unpaired *t*-test, *n* = 3). **g** The experimental scheme of fibroblasts challenged with different concentrations of migrasome inhibitor co-culture with macrophages using transwell inserts with a pore size of 3 µm. **h**, **i** Immunoblotting images (**h**) and analysis of the relative α-SMA protein expression in macrophages (i) which co-cultured with fibroblasts challenged with different concentrations of migrasome inhibitor SAR407899 using transwell inserts with a pore size of 3 µm (*P < 0.05, **P < 0.01, ***P < 0.001, one-way ANOVA, *n* = 3). **j** Confocal images of mitochondrial morphology (red) and dsDNA (green) in a L929 cell. Scale bar, 5 µm. **k** Quantification of extranuclear dsDNA foci per cell (****P < 0.0001, unpaired *t*-test, *n* = 50). **l** Confocal images of migrasomes with WGA staining (red) and dsDNA (green) in a L929 cell. Scale bar, 10 µm. **m** Quantitative analysis of dsDNA-positive migrasome number in each L929 cell (****P < 0.0001, unpaired *t*-test, *n* = 50). **n**, **o** Immunoblotting images (**n**) and analysis of the relative α-SMA protein expression (**o**) in macrophages after challenge by purified migrasomes from both control and LPS-stimulated fibroblasts (*P < 0.05, unpaired *t*-test, *n* = 4). **p** A schematic of fibroblast mitochondrial mtDNA isolation and transfection into macrophage. **q**, **r** Immunoblotting images (**q**) and analysis of relative α-SMA protein expression (**r**) in macrophages after transduction with mtDNA from control or LPS-stimulated fibroblast donors (*P < 0.05, unpaired *t*-test, *n* = 3). **s** Immunofluorescence microscopy image of TSPAN4-mCherry-labeled migrasomes derived from fibroblasts seeded in the upper chamber taken up by macrophages seeded in the lower chamber in a transwell setup with a pore size of 3 µm. The arrow indicates TSPAN4-mCherry-positive signal in the macrophages. Scale bar, 50 µm. **t** Quantification of the phagocytosis rate (****P < 0.0001, unpaired *t*-test, *n* = 6).

Our results demonstrate that LPS stimulation leads to an increased expression of migrasomes. Given that migrasomes are a novel medium for intercellular communication, we questioned whether they participate in the interaction between fibroblasts and macrophages, potentially promoting the MMT. To explore this possibility, and considering that migrasomes have an average diameter of 0.5–3 μm (ref. ^16^), we used a co-culture system of fibroblasts and macrophages using transwell inserts with two different pore sizes. Initially, we used transwell inserts with a pore size of 3 µm, which allows migrasome passage (Fig. 3a). To investigate whether LPS stimulation alone induces MMT, we established control experiments where macrophages were exposed to LPS in the absence of fibroblast-derived migrasomes using the same transwell setup. Specifically, we added DMEM medium containing LPS but without fibroblasts in the upper chamber. The results showed that LPS stimulation alone did not induce increased expression of α-SMA in macrophages. However, under the same conditions, where fibroblast-derived migrasomes could penetrate into the lower chamber, co-culturing macrophages with LPS-challenged fibroblasts led to a significant increase in macrophage α-SMA expression levels compared with the control group (Fig. 3b, c). By contrast, when using transwell inserts with a pore size of 0.4 µm, which block migrasomes from crossing into the lower chamber (Fig. 3d), the interactions between fibroblast-released migrasomes and macrophages were inhibited, and LPS had no effect on α-SMA expression levels in macrophages (Fig. 3e, f). In addition, we treated fibroblasts with the migrasome inhibitor SAR407899 (ref. ^29^) and used transwell inserts with a pore size of 3 µm (Fig. 3g). We found that, with a markedly reduced number of migrasomes, macrophage α-SMA expression levels were significantly inhibited compared with the control group, showing a significant correlation with the number of migrasomes formed (Fig. 3h, i). These results suggest that migrasomes are involved in the MMT process.

Next, we aimed to identify which components within migrasomes might induce the MMT phenomenon in macrophages. Previous studies have reported that damaged mitochondria can be disposed of via migrasomes through a process known as mitocytosis^17^. This suggests that cells can actively translocate cytosolic contents, such as mitochondria, into migrasomes, thereby releasing cytosolic material into the extracellular space. Migrasomes can subsequently be taken up by surrounding cells^16^. We hypothesized that LPS stimulation causes mitochondrial damage in fibroblasts, leading to the release of large amounts of mtDNA.

To verify this, we first examined the fibroblasts cultured under normal conditions, which displayed typical filamentous mitochondria with interconnected networks. However, upon LPS exposure, fibroblast mitochondria became fragmented and punctate. This morphological change led to a rapid and pronounced increase in mtDNA release into the cytosol, attributable to mitochondrial damage (Fig. 3j, k). To investigate whether mtDNA could be transported via migrasomes, we used immunofluorescence to explore the distribution of mtDNA and found a significant increase in extranuclear dsDNA co-localized with migrasomes in fibroblasts under LPS stimulation (Fig. 3l, m).

Considering the crucial role of mtDNA in fibrosis^30^, and to investigate the role of mtDNA migrasomes in fibroblast–macrophage interactions, we isolated migrasomes from fibroblasts in both the control and LPS-stimulated groups. To determine the optimal concentration of migrasomes for inducing MMT changes in macrophages, we applied a concentration gradient of purified migrasomes (0.1, 0.5, 1 and 2 µg). Our results revealed that 0.1 µg of migrasomes from LPS-stimulated fibroblasts induced the most significant MMT effects compared with untreated control macrophages (Supplementary Fig. 4b). We further assessed macrophage MMT phenotypic changes after treatment with 0.1 µg of migrasomes from both control and LPS-stimulated fibroblasts for 24 h. The results demonstrated that migrasomes derived from LPS-treated fibroblasts significantly upregulated α-SMA protein expression in macrophage (Fig. 3n, o). Similar to the effect of purified migrasomes, transfection of macrophage with mtDNA isolated from LPS-stimulated fibroblasts significantly upregulated α-SMA expression (Fig. 3p–r). Furthermore, fluorescence microscopy results confirmed that migrasomes labeled with TSPAN4-mCherry in the L929 cell line (Supplementary Fig. 4c), which were secreted by LPS-stimulated fibroblasts, were more efficiently phagocytosed by macrophages compared with those from the control group, as demonstrated using a transwell co-culture system (Fig. 3s, t). These data provide evidence that MMT is associated with the effects of LPS-challenged fibroblast-derived mtDNA migrasomes.

### PGC-1α activation alleviates LPS-induced mitochondrial dysfunction and suppresses the fibroblast mtDNA-migrasome formation

Mitochondrial dysfunction induced by LPS results in accumulation of mtDNA in the cytoplasm, from where overload mtDNA are disposed to extracellular by migrasomes. To further investigate the mechanism underlying the release of mtDNA migrasomes, PGC-1α as a key transcriptional co-activator of mitochondrial biogenesis, which is tightly related to mitochondria homeostasis and mtDNA replication^31^, has intrigued us. Therefore, to investigate whether improving mitochondrial function by activating PGC-1α could affect migrasome formation, we applied the pharmacological agent ZLN005 to activate PGC-1α (ref. ^32^). The concentration of 25 μM for ZLN005 was chosen on the basis of cytotoxicity results obtained from CCK-8 assays (Supplementary Fig. 5a). Our results show that, in LPS-challenged fibroblasts, PGC-1α levels are typically suppressed. However, treatment with ZLN005 not only elevated PGC-1α expression but also reduced α-SMA protein expression in these fibroblasts (Supplementary Fig. 5b). Moreover, PGC-1α activated alone in normal fibroblasts increased mitochondrial mass and reduced mtDNA release from damaged mitochondria induced by LPS (Supplementary Fig. 5c, d). Treatment with ZLN005 also resulted in decreased migrasome formation (Supplementary Fig. 5e, f).

**Fig. 4:**
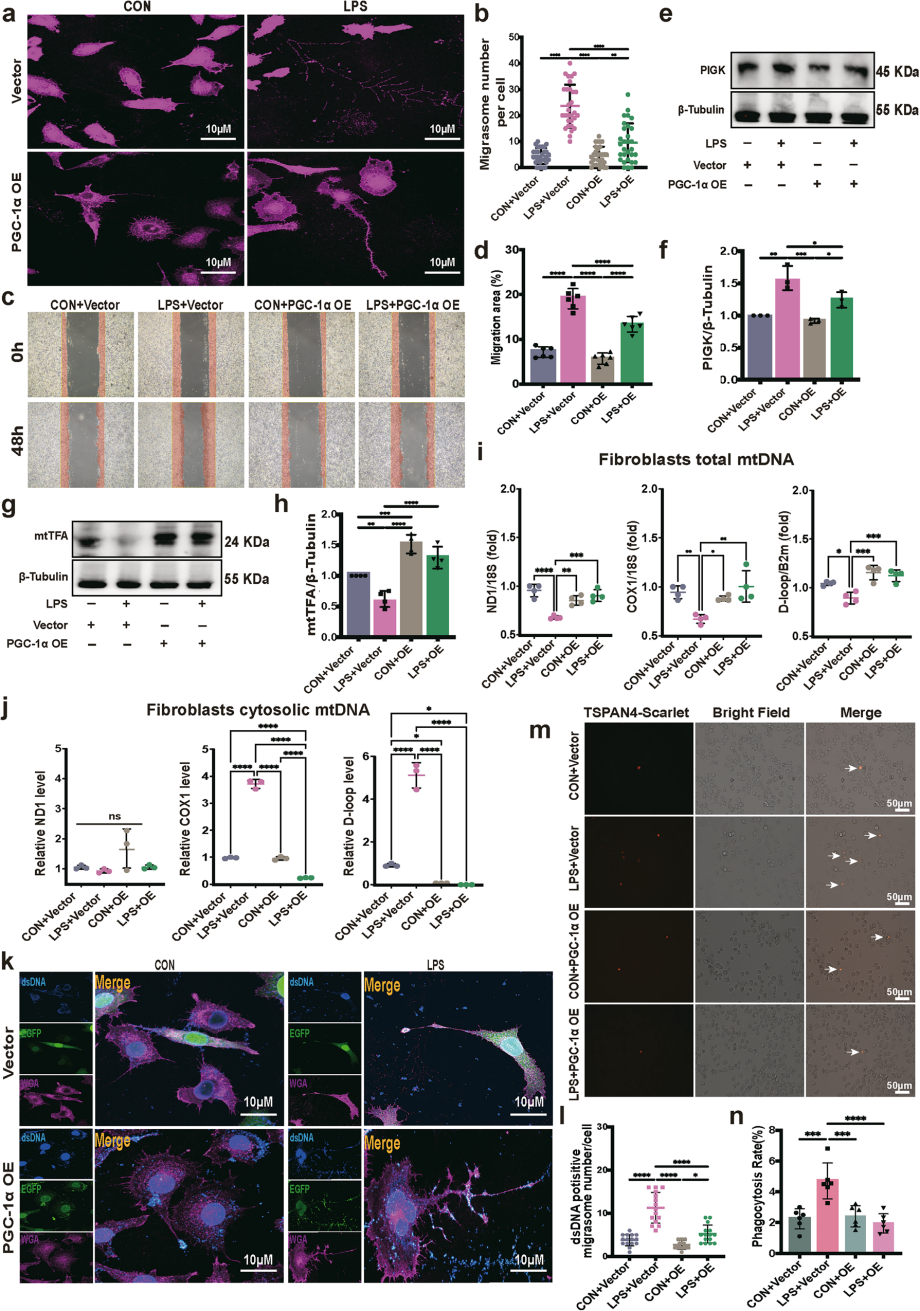
Activating PGC-1α alleviates LPS-induced mitochondrial dysfunction and suppresses the fibroblast mtDNA-migrasome formation. **a** Typical confocal images of PGC-1α-overexpressing (OE) or vector fibroblasts with or without LPS stimulation by WGA staining. Scale bar, 10 µm. **b** Statistics of migrasome number per cell in migrasome-containing fibroblasts (**P < 0.01, ***P < 0.001, ****P < 0.0001, one-way ANOVA, *n* = 30). **c**, **d** Microscopic inspection (**c**) of PGC-1α-overexpressing or vector fibroblasts with or without LPS stimulation after scratching (0 h) and after 48 h of wound healing. The bar plots (**d**) show the relative fibroblast migration area percentage (*P < 0.05, ****P < 0.0001, one-way ANOVA, *n* = 6). **e**, **f** Western blot images of migrasome marker protein PIGK relative protein expression from vector-transfected or PGC-1α-overexpressing transfected fibroblasts with or without LPS exposures (**e**) and quantified (**f**) (*P < 0.05, **P < 0.01, ***P < 0.001, one-way ANOVA, *n* = 3). OE means overexpression. **g**, **h** mtTFA protein expression of PGC-1α-overexpressing L929 cells or vector-transfected L929, and stimulated with or without LPS, detected using western blot (**g**) and quantified (**h**) (*P < 0.05, **P < 0.01 ***P < 0.001, one-way ANOVA, *n* = 4). OE means overexpression. **i** Relative total mtDNA amounts in stable PGC-1α-overexpressing L929 cells or vector-transfected L929 cells stimulated with or without LPS. The ratios of ND1 mtDNA to 18S nDNA, Cox1 mtDNA to 18S nDNA and D-loop mtDNA to B2M nDNA are shown (*P < 0.05, **P < 0.01, ***P < 0.001, ****P < 0.0001, one-way ANOVA, *n* = 4). OE means PGC-1α-overexpressing. **j** Relative cytosolic mtDNA amounts in PGC-1α-overexpressing or vector control fibroblasts stimulated with or without LPS (*P < 0.05, ****P < 0.0001, one-way ANOVA, *n* = 3). OE means PGC-1α-overexpressing. **k** Representative fluorescent confocal images of L929 cells stably overexpressing PGC-1α or vector-transfected L929 cells stimulated with or without LPS and co-stained for dsDNA (blue) and migrasome (fuchsia). EGFP (green) fluorescent signal from the transfected plasmid. Scale bar, 10 µm. **l** Quantitative analysis of dsDNA-positive migrasome number in each L929 cell (*P < 0.05, ****P < 0.0001, one-way ANOVA, *n* = 15). OE means overexpression. **m** Immunofluorescence microscopy images of TSPAN4-mScarlet-labeled migrasomes derived from PGC-1α-overexpressing or vector-transfected L929 fibroblasts seeded in the upper chamber, showing uptake by macrophage seeded in the lower chamber of a transwell setup with a pore size of 3 µm. The arrow indicates TSPAN4-mScarlet-positive signal in the macrophages. Scale bar, 50 µm. **n** Quantification of the phagocytosis rate (***P < 0.001, ****P < 0.0001, one-way ANOVA, *n* = 6).

To further confirm the relationship between PGC-1α expression and migrasome formation, we utilized a fibroblast cell line stably overexpressing PGC-1α (Supplementary Fig. 6a, b). Migrasome formation was assessed using WGA staining and migrasome-specific protein markers via western blot. We observed minimal formation of migrasomes on retraction fibers in PGC-1α-overexpressing fibroblasts even with LPS stimulation (Fig. 4a, b). Our findings indicated that fibroblast migration, evaluated by a wound healing assay, was notably reduced under PGC-1α overexpression conditions (Fig. 4c, d). Consistent with these observations, the upregulation of migrasome protein marker PIGK by LPS exposure was significantly reversed by PGC-1α overexpression (Fig. 4e, f).

Given the critical role of mitochondrial transcription factor A (mtTFA) in regulating mtDNA transcription, packaging and copy number^30^, we also examined mtTFA expression. We found that mtTFA expression was also significantly inhibited by LPS stimulation but upregulated by PGC-1α overexpression (Fig. 4g, h). Moreover, to further verify that overexpression of PGC-1α is beneficial to mitochondria function and inhibition of migrasome release, the relative mtDNA levels in the total cell and cytoplasm were measured. LPS-challenged fibroblasts exhibited reduced mtDNA in whole cells compared with the control group, a phenomenon reversed by PGC-1α overexpression (Fig. 4i). Furthermore, mtDNA leakage into the cytosol, significantly elevated by LPS stimulation compared with the control group, was abolished by PGC-1α overexpression (Fig. 4j). We also found that the dsDNA signal inside migrasomes was diminished by PGC-1α overexpression (Fig. 4k, l). To address whether PGC-1α overexpression in fibroblasts alters migrasome uptake by macrophages, we established TSPAN4-mScarlet-expressing fibroblast lines, including both PGC-1α-overexpressing and empty vector-transfected cells (Supplementary Fig. 6c). We noticed that, when macrophages were co-cultured with TSPAN4-Scarlet-labeled migrasomes from PGC-1α-overexpressing fibroblasts, there was a significant reduction in the uptake of migrasomes by macrophages compared with the vector control group under identical LPS stimulation conditions (Fig. 4m, n).

These results suggest that enhancing mitochondrial biogenesis via PGC-1α overexpression could prevent mtDNA leakage induced by LPS, thereby reducing the formation and need for migrasomes. This, in turn, leads to the significant decrease in phagocytosis of migrasome by macrophages.

### PGC-1α-mediated mtDNA-migrasome release from fibroblasts is required for MMT

**Fig. 5:**
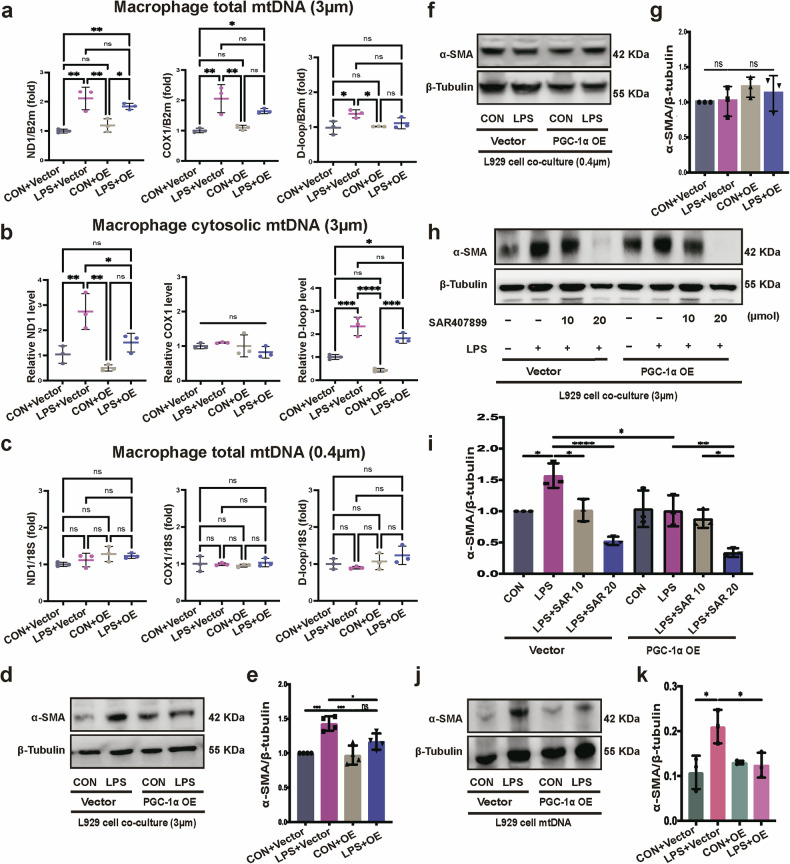
PGC-1α-mediated mtDNA-migrasome release from fibroblasts is required for MMT. **a** Relative total mtDNA amounts in macrophage co-cultured with PGC-1α-overexpressing fibroblasts or vector-transfected fibroblasts challenged with or without LPS using a transwell insert system with a pore size of 3 µm (*P < 0.05, **P < 0.01, one-way ANOVA, *n* = 3). **b** Relative cytosolic mtDNA amounts in macrophages co-cultured with PGC-1α-overexpressing fibroblasts or vector-transfected fibroblasts challenged with or without LPS using a transwell insert system with a pore size of 3 µm. The relative ratios of ND1 mtDNA, COX1 mtDNA and D-loop mtDNA are shown (*P < 0.05, ****P < 0.0001, one-way ANOVA, *n* = 3). **c** Relative total mtDNA amounts in macrophages co-cultured with PGC-1α-overexpressing fibroblasts or vector-transfected fibroblasts challenged with or without LPS using a transwell insert system with a pore size of 0.4 µm (one-way ANOVA, *n* = 3). **d**, **e** Immunoblotting images (**d**) and quantification analysis (**e**) of the α-SMA protein in macrophages co-cultured with PGC-1α-overexpressing fibroblasts or vector control fibroblasts with or without LPS stimulation while migrasome gets access to macrophage in the lower chamber (transwell inserts with a pore size of 3 µm) (*P < 0.05, **P < 0.01, ***P < 0.001, one-way ANOVA, *n* = 4). **f**, **g** Immunoblotting images (**f**) and quantification analysis (**g**) of the α-SMA protein in macrophages co-cultured with PGC-1α-overexpressing fibroblasts or vector control fibroblasts with or without LPS stimulation while migrasome was considered impassable (transwell inserts with a pore size of 0.4 µm) (*n* = 3). **h**, **i** Immunoblotting images (**h**) and quantification analysis (**i**) of the α-SMA protein in macrophages co-cultured with PGC-1α-overexpressing fibroblasts or vector control fibroblasts challenged with different concentrations of migrasome inhibitor SAR407899 using transwell inserts with a pore size of 3 µm (*P < 0.05, **P < 0.01, ****P < 0.0001, one-way ANOVA, *n* = 3). **j**, **k** Immunoblotting images (**j**) and quantification analysis (**k**) of the α-SMA protein in macrophages transduced with the same dosage of mtDNA donated from PGC-1α-overexpressing fibroblasts or vector control fibroblasts stimulated with or without LPS (*P < 0.05, one-way ANOVA, *n* = 3).

To explore the impact of PGC-1α overexpression in fibroblasts on the MMT, PGC-1α-overexpressing fibroblasts were co-cultured with macrophages, and changes in macrophage mtDNA levels were assessed. The results revealed that, while LPS-treated fibroblasts induced an increase in total mtDNA expression within macrophages, fibroblasts overexpressing PGC-1α under LPS stimulation showed heightened mtDNA expression compared with controls but a slight reduction in mtDNA levels in macrophages relative to the LPS-treated group (Fig. 5a). In addition, the cytoplasmic mtDNA content in macrophages mirrored the overall mtDNA expression trends (Fig. 5b). However, upon neutralizing the effects of migrasomes on macrophages, no significant differences in mtDNA expression were observed (Fig. 5c). These results suggest that reduced macrophage mtDNA levels after co-culture with PGC-1α-overexpressing cells are probably due to diminished migrasome uptake by macrophage. To assess whether the mtDNA content in migrasomes was altered, quantitative PCR (qPCR) analysis was performed on purified migrasomes. The results showed no significant differences in mtDNA levels in migrasomes derived from PGC-1α-overexpressing fibroblasts compared with controls (Supplementary Fig. 6d). However, the process of migrasome isolation proved labor intensive, requiring approximately 30 fibronectin-coated 15-cm culture dishes to obtain a single purified sample per group. This limitation in sample size hindered the ability to draw definitive conclusions regarding potential changes in migrasome mtDNA content. Despite these constraints, qPCR analysis confirmed the presence of both mtDNA and nuclear DNA within migrasomes. In summary, the reduction in macrophage mtDNA levels observed in this study is closely associated with decreased migrasome uptake. Whether changes in the mtDNA content of migrasomes also contribute to this effect requires further investigation.

To further elucidate the role of PGC-1α-overexpressing fibroblasts in modulating extracellular mtDNA transport and its influence on macrophage-to-myofibroblast transformation, a co-culture system was used. Under LPS stimulation, fibroblasts overexpressing PGC-1α released mtDNA via migrasomes, which had a subdued effect on macrophages compared with the vector-transfected fibroblast co-culture, leading to reduced α-SMA expression in macrophages (Fig. 5d, e). Furthermore, when migrasome function was inhibited, the influence of PGC-1α-overexpressing fibroblasts appeared diminished, as macrophages did not show signs of transitioning into myofibroblasts (Fig. 5f–i).

To validate these findings, equivalent doses of mtDNA extracted from fibroblasts subjected to various stimuli were transfected into macrophages. Notably, mtDNA from LPS-treated fibroblasts prompted an upregulation in α-SMA expression in macrophages, while exposure to mtDNA from PGC-1α-overexpressing fibroblasts after LPS treatment led to attenuated α-SMA expression (Fig. 5j, k). These results demonstrate that PGC-1α overexpression reduces the profibrotic impact of fibroblast-derived mtDNA migrasomes on macrophages.

### PGC-1α activation mitigates LPS-associated pulmonary fibrosis by inhibiting the MMT process

To further ascertain whether the activation of PGC-1α in pulmonary tissue can inhibit MMT and thereby delay the progression of SAPF, mice received intratracheal administration with PGC-1α-overexpressing AAV. Three weeks after administration, the mice were challenged with LPS (Supplementary Fig. 7a). The effectiveness of PGC-1α-overexpressing AAV was measured by immunofluorescence (Supplementary Fig. 7b) and western blot (Supplementary Fig. 7c).

**Fig. 6:**
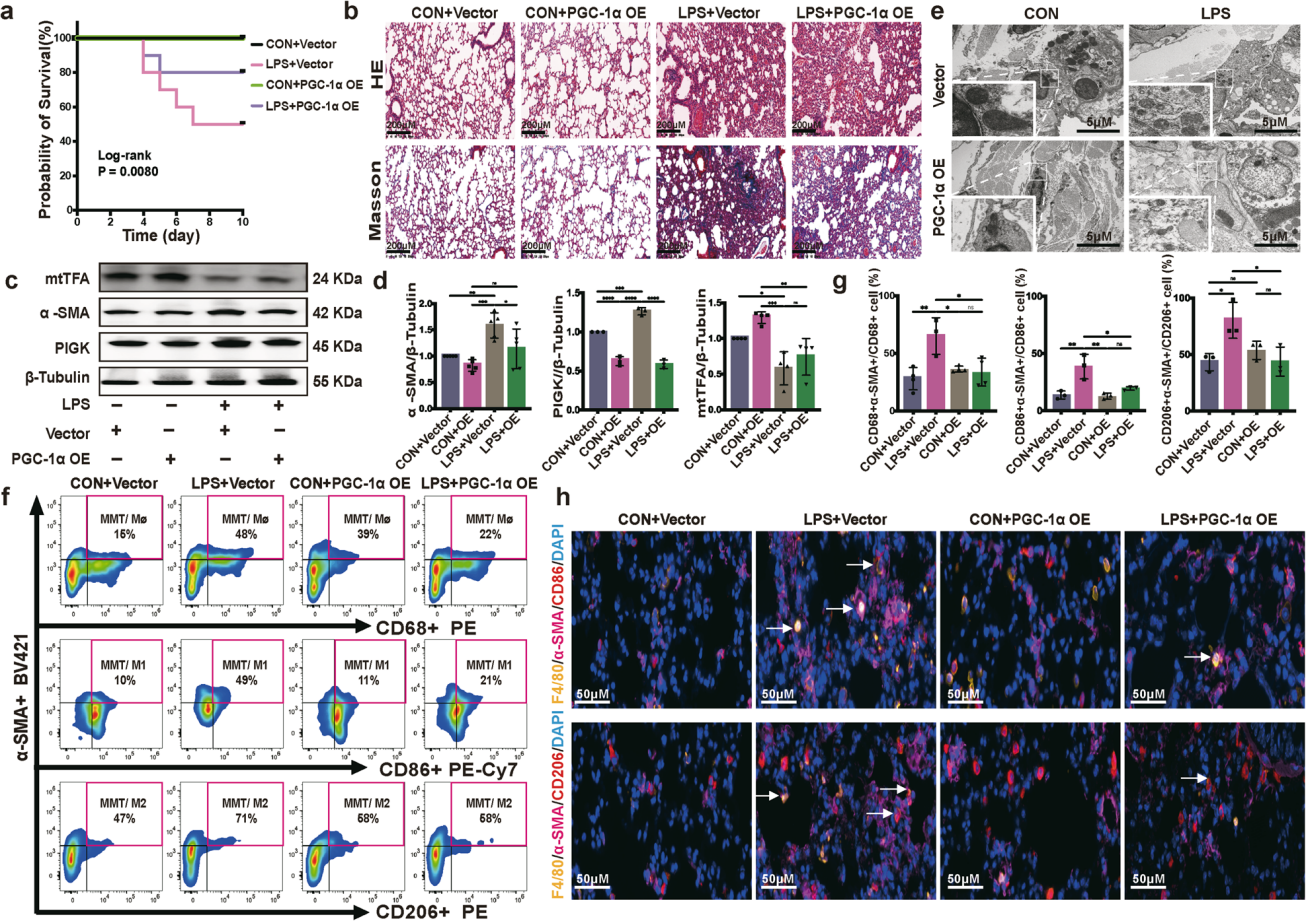
PGC1α activation alleviates LPS-associated pulmonary fibrosis by restraining the MMT process. **a** Kaplan–Meier survival curve of PGC-1α-overexpressing or vector-transfected mice after LPS or saline administration (log-rank (Mantel–Cox) test, *n* = 10 mice). **b** Typical images of H&E (top) and Masson’s trichrome (bottom) staining of PGC-1α-overexpressing AAV or vector-transfected mouse lung sections. Scale bar, 200 µm. **c**, **d** mtTFA, α-SMA and PIGK protein expression of PGC-1α-overexpressing or vector-transfected mouse lung tissue with or without LPS exposure detected using western blot (**c**), followed by quantification analysis (**d**) (*P < 0.05, **P < 0.01, ***P < 0.001, ****P < 0.0001, one-way ANOVA, *n* = 3 for PIGK, *n* = 4 for mtTFA, *n* = 5 for α-SMA). **e** TEM images of the mouse lung fibroblast mitochondria. PGC-1α overexpression in SAPF mouse lung fibroblasts significantly alleviated the mitochondria damage compared with vector-pretreated SAPF mice. The enlarged image shows mitochondria in fibroblasts. Scale bar, 5 µm. **f** MMTs (α-SMA^+^ CD68^+^), M1-MMTs (α-SMA^+^ CD86^+^) and M2-MMTs (α-SMA^+^ CD206^+^) in murine lung quantifyied by flow cytometry analysis. Gated on CD11b^+^ F4/80^+^ macrophages. **g** Bar plots representing the percentage of α-SMA^+^ CD68^+^ cells to CD68^+^ cells, α-SMA^+^ CD86^+^ cells to CD86^+^ cells and α-SMA^+^ CD206^+^ cells to CD206^+^ cells respectively (*P < 0.05, **P < 0.01, one-way ANOVA, *n* = 3, *n* = 4 for the CON+vector group and the CON + OE group of CD68^+^ analysis). **h** Multiplex immunofluorescence enlarged merged images of F4/80^+^ CD86^+^ α-SMA^+^ cells (top) and F4/80^+^ CD206^+^ α-SMA^+^ cells (bottom) in mouse lung tissues. Scale bar, 50 μm.

Body weight was monitored from the first day of LPS administration as the baseline, and subsequent days, with the body weight change curve shown in Supplementary Fig. 7d. Notably, LPS treatment caused significant weight loss in the LPS+vector group, with the lowest weight observed on day 4. In the LPS+OE group, “OE” refers to PGC-1α-overexpressing group, weight loss was mitigated, while the CON+vector and CON+OE groups, “CON” refers to control group, showed no initial body weight loss. Consistently, as shown in Fig. 6a, PGC-1α overexpression significantly improved the survival rate of mice under LPS stress. PGC-1α overexpression alleviates LPS-induced weight loss and mortality rate, highlighting its protective role against inflammatory stress and its potential as a therapeutic target for sepsis-related conditions. Moreover, PGC-1α overexpression markedly reduced extracellular matrix deposition and inflammatory reaction (Fig. 6b), accompanied by a downregulation of α-SMA and PIGK protein expression and an upregulation of mtTFA protein and total mtDNA levels (Fig. 6c, d and Supplementary Fig. 7e) in mouse pulmonary tissue. TEM results further revealed that, compared with control mice, pulmonary fibroblasts in the LPS-treated group displayed typical signs of mitochondrial damage, such as swelling and loss of inner mitochondrial membrane integrity and cristae, which were significantly alleviated in the PGC-1α overexpression pretreatment group (Fig. 6e).

To further confirm that PGC-1α mediates MMT in vivo, impacting LPS-induced pulmonary fibrosis, we assessed MMT cells using flow cytometry analysis and immunofluorescence staining. As expected, MMT cells (α-SMA^+^ CD68^+^) were decreased significantly in PGC-1α-overexpressing mice, with both M1-MMT (α-SMA^+^ CD86^+^) and M2-MMT (α-SMA^+^ CD206^+^) populations being significantly reduced in the SAPF model (Fig. 6f, g). Furthermore, multiplex immunofluorescence imaging results further confirmed these changes in MMT cells in SAPF (Fig. 6h and Supplementary Fig. 7f, g).

Therefore, these results demonstrate that PGC-1α overexpression inhibits mtDNA-migrasome secretion, thereby restraining the transition of macrophages to myofibroblasts and, consequently, alleviating SAPF.

## Discussion

The lungs are particularly vulnerable during sepsis, characterized by increased vascular permeability, inflammatory cell infiltration and oxidative stress^33^. This often leads to the activation and differentiation of fibroblasts into myofibroblasts, which are key contributors to the excessive deposition of extracellular matrix in the lungs. Despite ongoing research, the origins and mechanisms of fibroblast formation within pulmonary fibroblast foci remain critical areas of study in SAPF pathogenesis^4,31,34^. Our study presents both in vivo and in vitro evidence demonstrating that migrasomes containing mtDNA, secreted by lung fibroblasts in response to LPS-induced migration and mitochondrial dysfunction, facilitate the MMT. This process promotes the formation of pulmonary fibroblast foci and advances SAPF progression. Furthermore, the inhibition of fibroblast PGC-1α by LPS plays a substantial role in these processes, offering new insights into SAPF mechanisms and suggesting potential therapeutic targets.

Extracellular vesicles are increasingly recognized for their role in intercellular communication in fibrotic diseases^35^. Migrasomes, a novel intercellular communication medium formed by the release of cytoplasmic contents from retraction fibers, have been implicated in various pathological processes^16^. Although their precise roles are not fully elucidated, studies indicate that migrasomes can enhance migration and proliferation when internalized by retinal pigmented epithelium, exacerbating proliferative vitreoretinopathy^18^. Furthermore, migrasomes derived from monocytes are crucial for angiogenesis, delivering angiogenic factors to the capillary formation microenvironment^36^. Previous research highlights the importance of mitocytosis—a process of damaged mitochondria disposal by migrasomes—in maintaining mitochondrial quality and cellular homeostasis by expelling damaged mitochondria^17^. Our study suggests that accumulated mtDNA in cytoplasm was released extracellularly via migrasomes to maintain cellular homeostasis. Migrasomes serve as key transport vehicles in this process. Notably, our results show that LPS stimulation significantly increases migrasome release from fibroblasts. Furthermore, mtDNA from fibroblasts overexpressing PGC-1α exhibits a diminished capacity to induce MMT, indicating that the impact of migrasomes on MMT depends on both the quantity and specific properties of mtDNA. Further research is required to elucidate the specific roles of migrasomes in SAPF and fibroblast activation, including characterizing migrasome contents released by activated fibroblasts, their effects on other cell types in the fibrotic microenvironment and their potential as therapeutic targets.

The presence and extent of fibroblast foci in SAPF may have prognostic implications, as the number and size of these fibroblast foci correlate with disease severity and poor outcomes in patients with idiopathic pulmonary fibrosis^37^. Similarly, pulmonary fibrosis development in sepsis survivors is linked to increased morbidity and mortality^38^. MMT has emerged as a substantial cellular process contributing to fibrosis, involving the phenotypic and functional conversion of macrophages into myofibroblasts, the primary cells responsible for excessive extracellular matrix protein deposition in fibrotic diseases^9,39^. MMT serves as a crucial link between inflammation and fibrosis in various organ systems, including the lungs, liver and kidneys^10,11,40,41^. Moreover, MMT also contributed to fibroblast foci development in proliferative vitreoretinal disorders and intestinal fibrosis^12,42^. The inflammatory milieu, characterized by the presence of other cytokines and growth factors, also supports the MMT by inducing epigenetic modifications and transcriptional changes in macrophages that favor a myofibroblast-like phenotype^43^. Our study provides confirmation of the MMT process in SAPF, showing that both M1 macrophages and M2 macrophages promote fibroblast accumulation through MMT. Interestingly, PGC-1α overexpression reduced the percentages of M1-MMT and M2-MMT in mice with alleviated lung fibrosis, challenging the conventional understanding that M1 macrophages have antifibrotic effects while M2 macrophages drive fibrosis^44^. This dual role of macrophages in SAPF highlights the complexity of macrophage plasticity and suggests that targeting both M1 and M2 macrophages could be beneficial in treating SAPF.

PGC-1α is pivotal for maintaining mitochondrial homeostasis by regulating genes involved in mtDNA replication, respiratory chain complexes and antioxidant defense^45^. It coordinates with nuclear respiratory factors (NRF1 and NRF2) and TFAM to promote mitochondrial biogenesis, enhancing cellular energy production and reducing reactive oxygen species accumulation^46^. Dysregulation of PGC-1α expression has been linked to mitochondrial dysfunction, a precursor to increased oxidative stress, and cellular damage. Recent studies have shown that PGC-1α downregulation leads to mitochondrial dysfunction and increased profibrogenic factors in renal fibrosis^47^ and suppresses hepatic stellate cell activation in liver fibrosis^48^. Our data reveal that PGC-1α levels are suppressed in fibroblasts after LPS simulation, accompanied by mitochondria dysfunction and cytoplasmic mtDNA accumulation, suggesting that mtDNA is relevant to innate immune responses and inflammation^22,49^. Furthermore, mitochondrial dysfunction can lead to cell death, further contributing to the fibrotic response through the release of profibrotic cytokines and growth factors^50,51^. Our results indicate that PGC-1α plays a critical role in this process by promoting migrasome formation, which facilitates the communication necessary for macrophages to transition into myofibroblasts, thus accelerating fibrosis. This insight into the role of PGC-1α presents a new target for therapeutic strategies aimed at mitigating SAPF.

In conclusion, our study reveals that LPS-induced pulmonary fibrosis involves the MMT, facilitated by fibroblast-derived migrasomes containing mtDNA. LPS stimulation enhances fibroblast migration and promotes the release of migrasomes, which transfer mtDNA to macrophages. This mtDNA-migrasome uptake induces macrophage differentiation into myofibroblasts, contributing to fibrosis progression. The activation of PGC-1α ameliorates LPS-induced mitochondrial dysfunction, suppresses the formation of mtDNA-migrasomes, inhibits the MMT process and, consequently, attenuates pulmonary fibrosis. Future research should focus on elucidating the precise molecular mechanisms by which mtDNA within migrasomes induces MMT in macrophages. Investigating the regulatory pathways of PGC-1α in controlling migrasome biogenesis and mtDNA release may uncover novel therapeutic targets. Furthermore, exploring the clinical potential of PGC-1α activators or migrasome inhibitors could lead to effective interventions for SAPF.

## Supplementary information

Supplementary Information

## Data Availability

Data are available from the authors upon request.
