# Supplementary material for: PGC-1α mediates migrasome secretion accelerating macrophage–myofibroblast transition and contributing to sepsis-associated pulmonary fibrosis
Source: Exp Mol Med. 2025 Apr 1;57(4):759–74. doi: 10.1038/s12276-025-01426-z (PMC12046055; doi:10.1038/s12276-025-01426-z)
Supplement: Supplementary file 1 — Supplementary Information [file 12276_2025_1426_MOESM1_ESM.pdf]

Supplementary figure legends

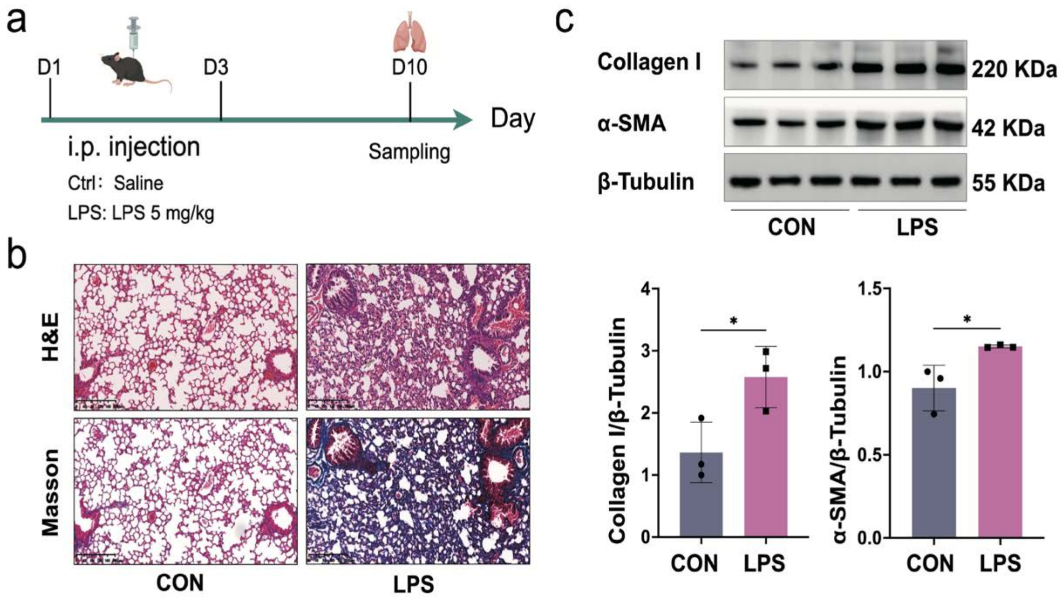

Supplementary Fig. 1

Sepsis-associated pulmonary fibrosis was induced in mice by LPS and fibroblasts were significant activated with LPS stimulation.

Intraperitoneal injection of 5mg/kg LPS for three consecutive days mouse model was used to investigate sepsis-associated pulmonary fibrosis. Histological analysis of lung tissues from mice in the LPS group revealed fibrosis, as evidenced by H&E and Masson staining, along with increased collagen I and α-SMA expressions.

a: A schematic of mouse model of sepsis-associated pulmonary fibrosis

b: Typical images of H&E (upper) and Masson's trichrome-stained (lower) mouse lung sections. Scale bar = 200 μm.

c: Collagen I and α-SMA protein expression in lung tissue were determined using western blot. Bar plots representing the relative expression (*$p < 0.05$, unpaired t-test, n = 3).

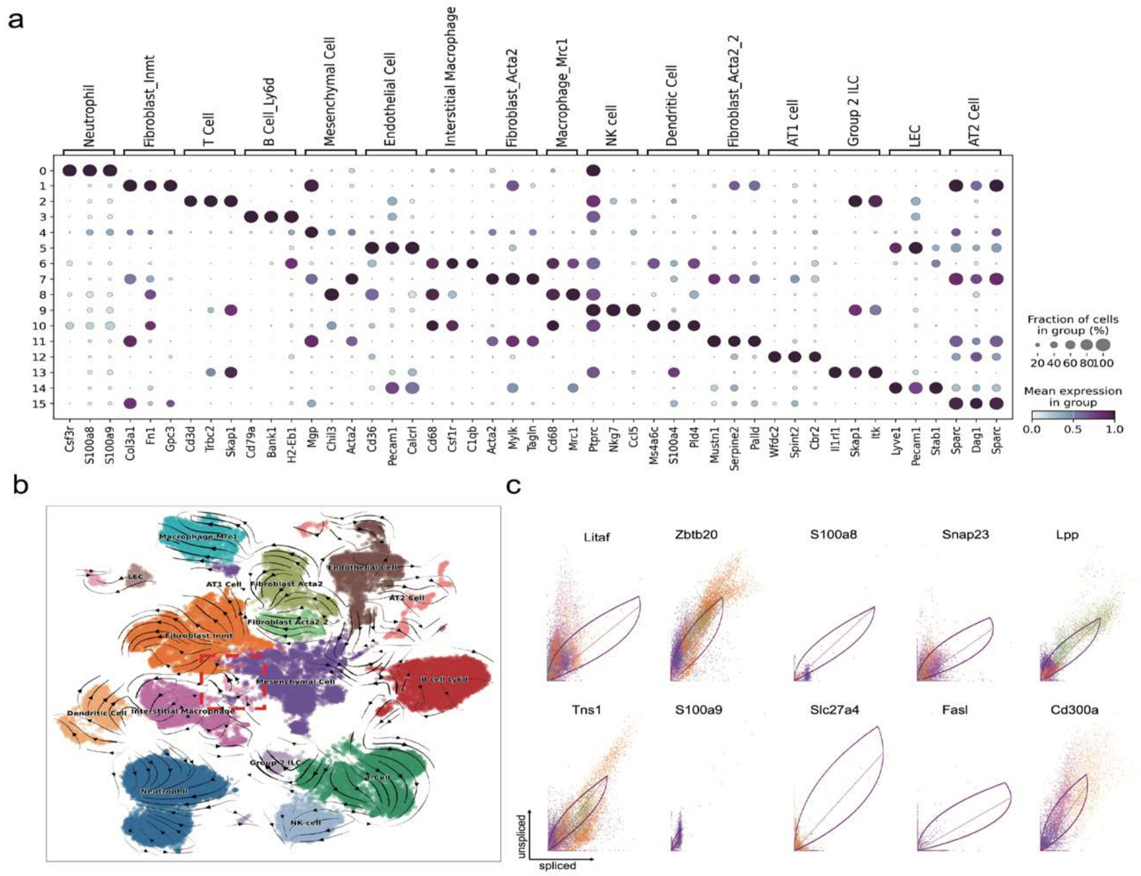

Supplementary Fig. 2

Clusters identification and RNA velocity analysis in mice lung tissue

a: Cell clusters identification marker gene are shown using dotplot. Dotplot of each cluster top three marker genes. The size of each circle depicts the percentage of cells in the subtype in which the marker was detected, and its color depicts the average transcript count in expressing cells.

b: RNA velocity analysis of single-cell RNA sequencing (scRNA-seq) data from all samples. Red frame highlights the trajectory of macrophage transformed into fibroblasts.

c: Scatter plot of spliced/unspliced mRNA counts of TOP 15 driver gene in different cell types.

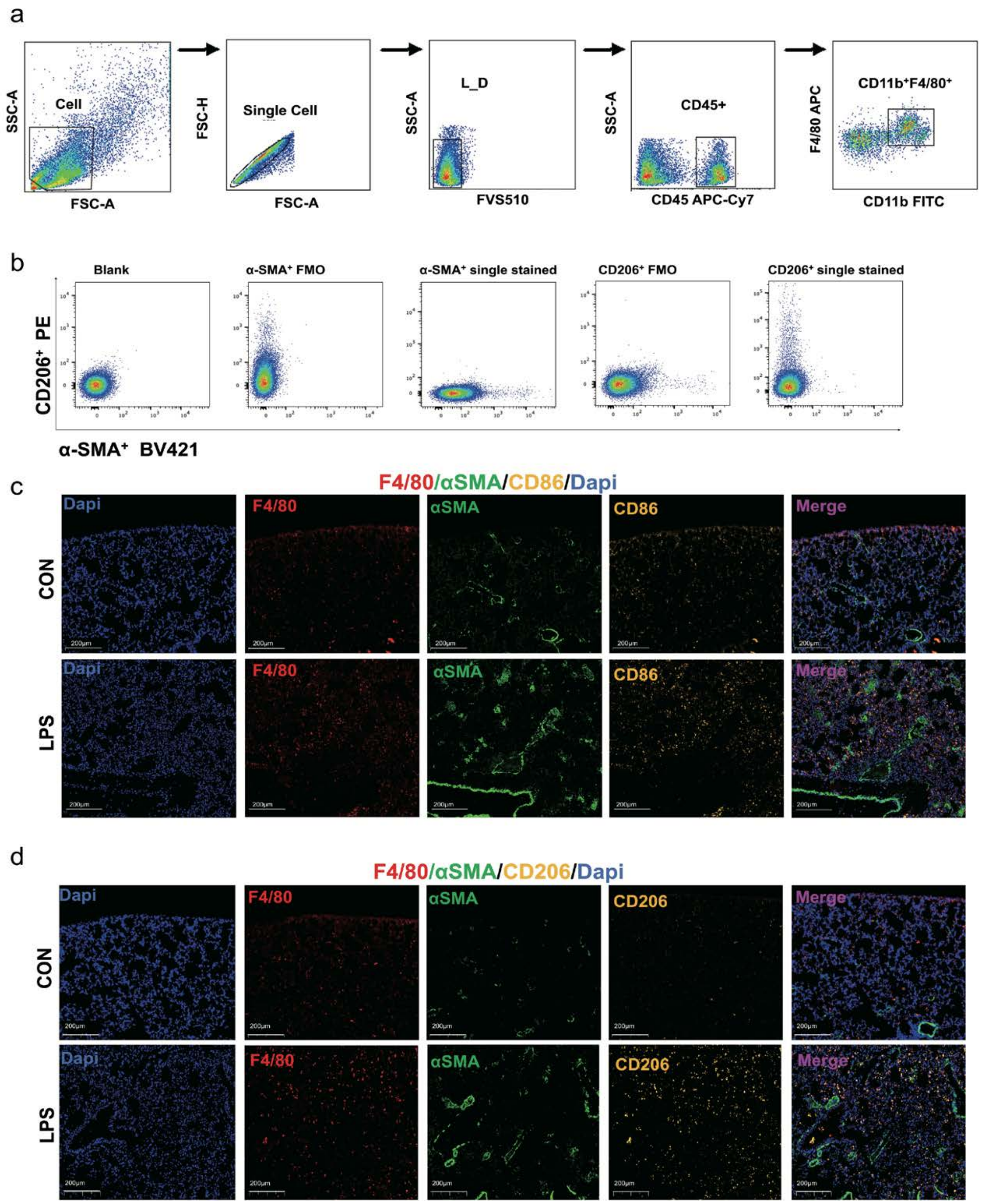

Supplementary Fig. 3

Identification of MMTs in mice lung tissues by flow cytometry and immunofluorescence.

a: Gating strategy of CD11b+/F4/80 positive macrophage in mice lung tissues. Cells were isolated from enzymatically digested mouse lungs, and after the exclusion of doublets and debris, live immune cells were identified by CD45 staining.

b: Blank, Fluorescence Minus One (FMO) control, and single stained image of α-SMA$^+$ cells or CD206$^+$ cells by flow cytometry.

c: Multiplex immunofluorescence images of F4/80 (red), CD86 (yellow), α-SMA (green), and DAPI for nuclear (blue) in mouse lung tissues. Scale bar = 200 μm.

d: Multiplex immunofluorescence images of F4/80 (red), CD206 (yellow), α-SMA (green), and DAPI for nuclear (blue) in mouse lung tissues. Scale bar= 200 μm.

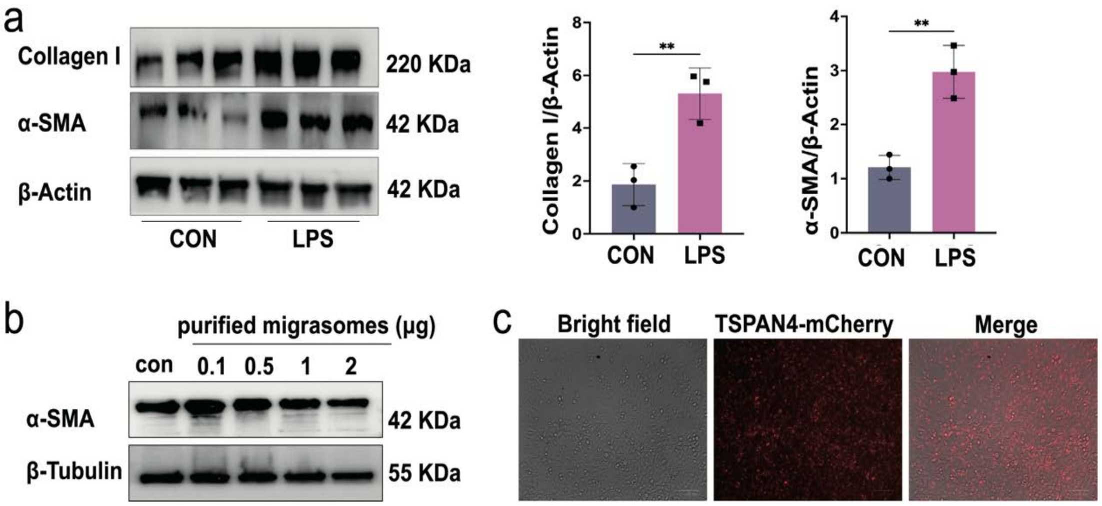

Supplementary Fig. 4

LPS stimulation activates fibroblasts migrasomes formation

a: Collagen I and α-SMA protein expression in L929 cells were determined using western blot. Bar plots representing the relative expression (*$p < 0.05$, **$p < 0.01$, unpaired *t*-test, n = 3).

b: α-SMA protein expression of macrophage that challenged with four concentration gradients of purified migrasome from LPS-stimulated fibroblasts.

c: Fluorescence image of TSPAN4-mCherry L929 stably expressing cell lines.

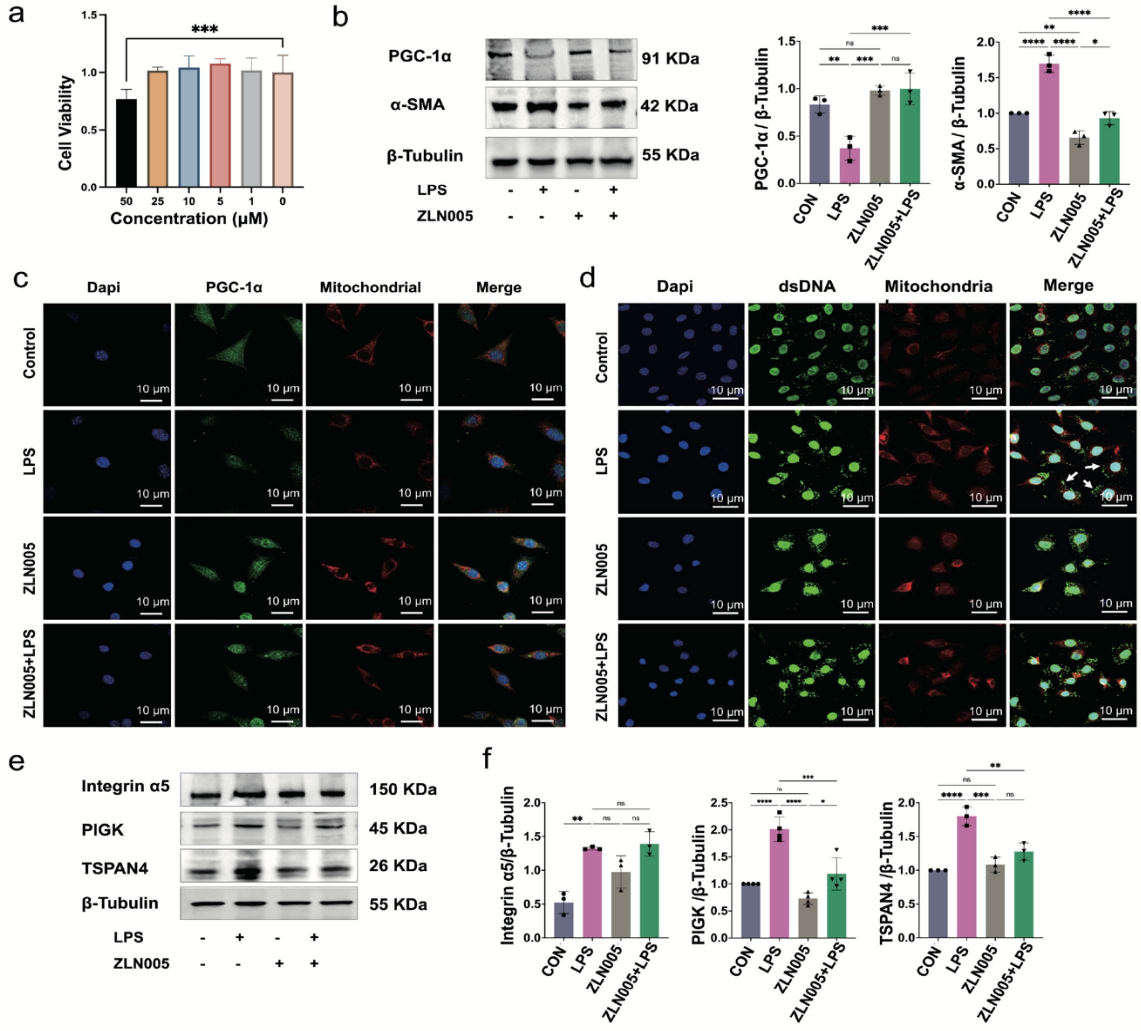

Supplementary Fig. 5

Activating PGC-1α by ZLN005 remains mitochondria homeostasis and inhibits migrasome formation and fibroblasts activation

a: The cytotoxic effects of ZLN005 at the indicated concentrations were determined by a CCK8 cell viability assay. Concentration of 25 μM ZLN005 was chosen based on CCK8 results (***$p < 0.001$, one-way ANOVA, n = 6)

b: Activated PGC-1α by ZLN0005 in fibroblasts effectively reduced the expression of cellular α-SMA (*$p < 0.05$, **$p < 0.01$, ***$p < 0.001$, ****$p < 0.0001$, one-way ANOVA, ns = not significant, n = 3).

c: Immunofluorescent staining was performed on fibroblasts pretreated with or without ZLN005 or LPS to evaluate PGC-1α expression (green) and mitochondria morphology changes (red). Cells nuclear was stained with DAPI (blue). Scale bar = 10 μm.

d: Representative fluorescent microscopy images of fibroblasts triple-labeled with mitotracker (red), dsDNA (green) and dapi (blue) followed by LPS or ZLN005 stimulation. Scale bar = 10 μm.

e and f: Integrin α5, PIGK and TSPAN4 protein expression of fibroblasts challenged with ZLN005 were detected by using western blot (**p* < 0.05, ***p* < 0.01, ****p* < 0.001, *****p* < 0.0001, one-way ANOVA, n = 3 for Integrin α5 and TSPAN4, n = 4 for PIGK)

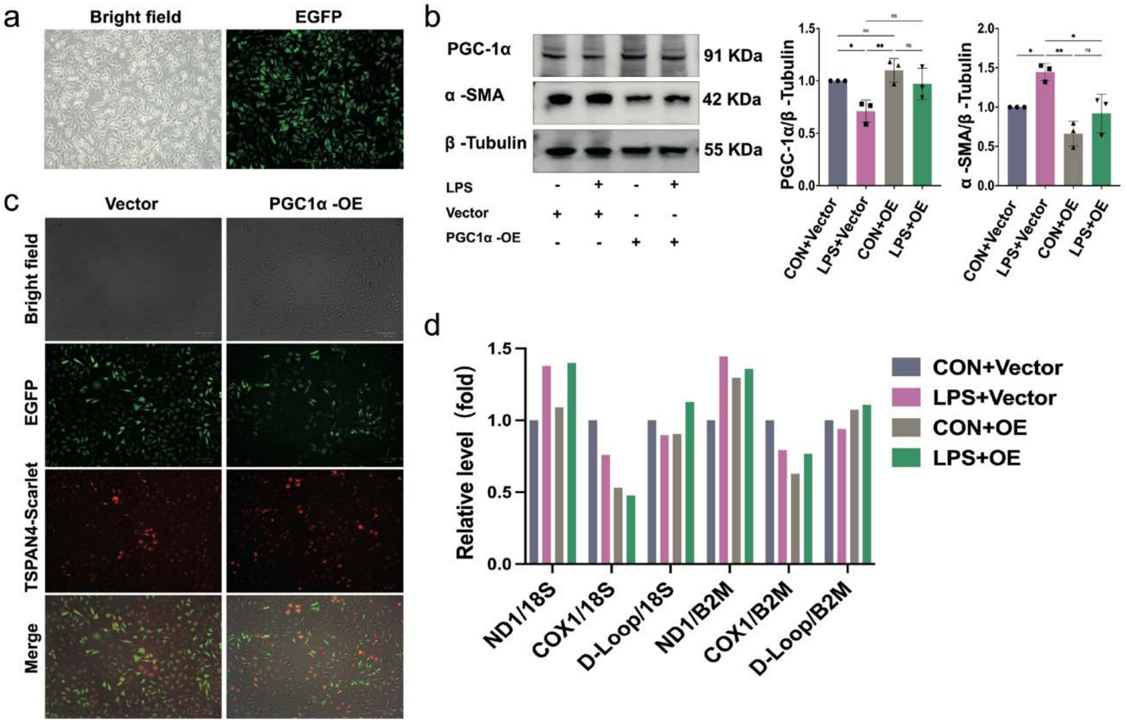

Supplementary Fig. 6

Overexpression PGC-1α in L929 fibroblasts reverse fibroblasts activation inducing by LPS.

a: PGC-1α OE lentivirus transfect efficacy detected by immunofluorescence. Bright field (left) and immunofluorescent image (right).

b: The efficacy of PGC-1α overexpression and fibroblasts activation were detected by western blot (**p* < 0.05, ****p* < 0.01, one-way ANOVA, n = 3).

c: Efficacy of TSPAN4-mScarlet lentivirus transfected into PGC-1α vector and PGC-1α OE L929 cell line were detected by immunofluorescence.

d: PCR analysis of mtDNA expression of purified migrasomes from one sample for each group.

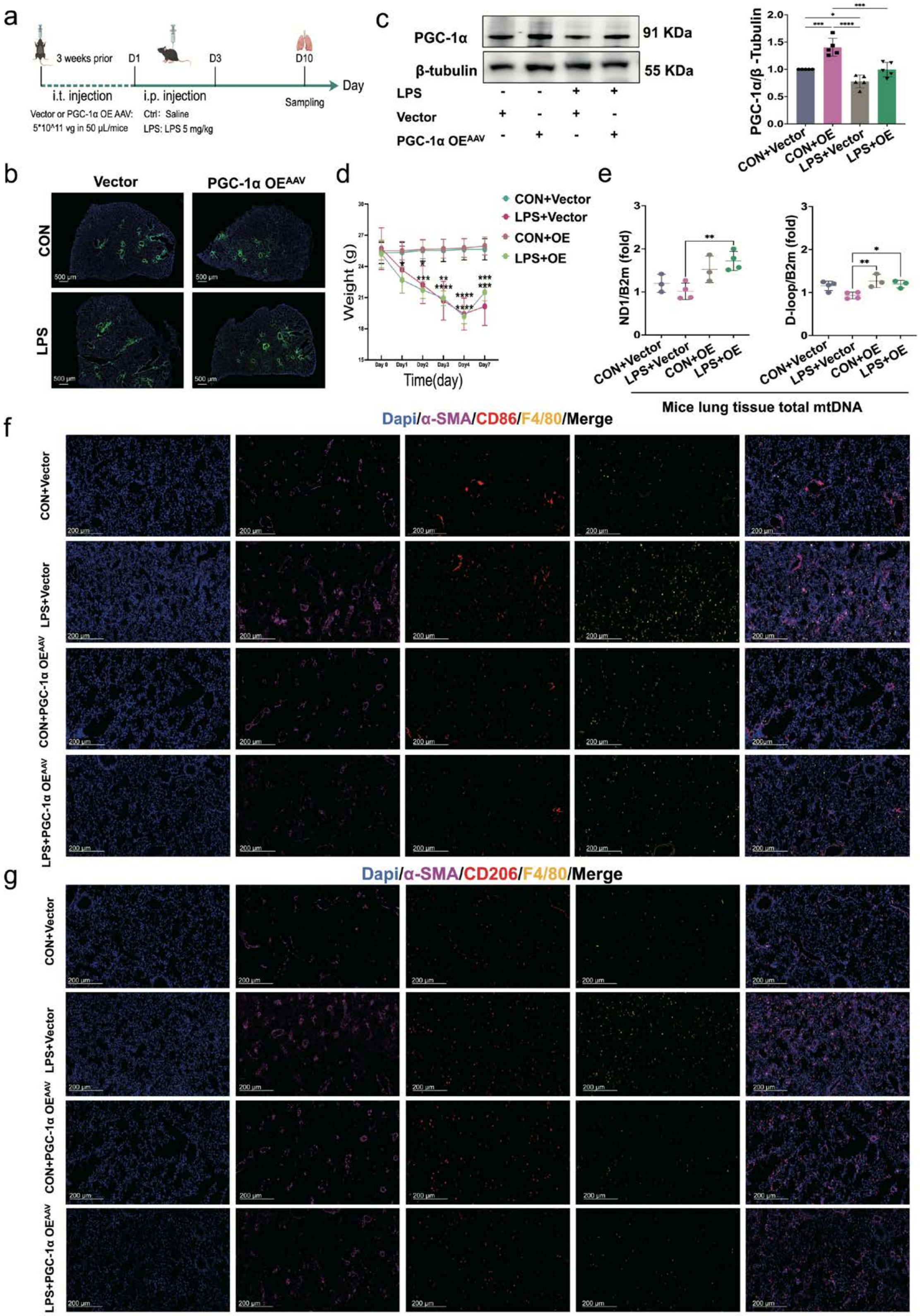
a
3 weeks prior
D1
D3
D10
Day
i.t. injection
i.p. injection
Sampling
Vector or PGC-1α OE AAV:
5*10^11 vg in 50 μL/mice
Ctrl: Saline
LPS: LPS 5 mg/kg
b
Vector
PGC-1α OE^AAV
CON
LPS
500 μm
c
PGC-1α
91 KDa
β-tubulin
55 KDa
LPS
Vector
PGC-1α OE^AAV
PGC-1α/β -Tubulin
CON+Vector
CON+OE
LPS+Vector
LPS+OE
d
CON+Vector
LPS+Vector
CON+OE
LPS+OE
Weight (g)
Time(day)
e
ND1/B2m (fold)
D-loop/B2m (fold)
Mice lung tissue total mtDNA
f
Dapi/α-SMA/CD86/F4/80/Merge
CON+Vector
LPS+Vector
CON+PGC-1α OE^AAV
LPS+PGC-1α OE^AAV
200 μm
g
Dapi/α-SMA/CD206/F4/80/Merge

Supplementary Fig. 7

Up-regulating PGC-1α inhibits LPS associate pulmonary fibrosis process in mice

a: Schematic demonstrates PGC-1α overexpressed mouse model procedure. Intratracheal injection of PGC-1α overexpression adeno-associated virus (AAV) or vector-AAV 3 weeks prior to LPS or saline intraperitoneal injection for 3 consecutive days.

b: Intratracheal injection of PGC-1α overexpression adeno-associated virus (AAV) or vector-AAV transfection efficiency was confirmed by fluorescence microscope detecting GFP signal.

c: The efficiency of PGC-1α overexpression was confirmed by immunoblotting analysis. (*$p < 0.05$, **$p < 0.01$, one-way ANOVA, n = 5).

d: Body weight change curve of PGC-1α OE or vector control mice challenged with or without LPS administration. (*$p < 0.05$, **$p < 0.01$, ***$p < 0.001$, ****$p < 0.0001$, two-way ANOVA, n = 6. CON+ Vector versus LPS+ Vector; CON+ OE versus LPS+ OE).

e: Relative total mtDNA amounts in mice lung tissues with overexpression PGC-1α or vector-transfected challenged with or without LPS (*$p < 0.05$, **$p < 0.01$, one-way ANOVA, n = 3 or 4).

f: Multiplex immunofluorescence images of F4/80 (yellow), CD86 (red), α-SMA (fuchsia), and dapi (blue) in mouse lung tissues with overexpression PGC-1α or vector-transfected. Scale bar = 200 µm.

g: Multiplex immunofluorescence images of F4/80 (yellow), CD206 (red), α-SMA (fuchsia), and dapi (blue) in mouse lung tissues with overexpression PGC-1α or vector-transfected. Scale bar = 200 µm.
